# Lateral and End-On Kinetochore Attachments Are Coordinated to Achieve Bi-orientation in *Drosophila* Oocytes

**DOI:** 10.1371/journal.pgen.1005605

**Published:** 2015-10-16

**Authors:** Sarah J. Radford, Tranchau L. Hoang, A. Agata Głuszek, Hiroyuki Ohkura, Kim S. McKim

**Affiliations:** 1 Waksman Institute of Microbiology, Rutgers, The State University of New Jersey, Piscataway, New Jersey, United States of America; 2 Department of Genetics, Rutgers, The State University of New Jersey, Piscataway, New Jersey, United States of America; 3 The Wellcome Trust Centre for Cell Biology, School of Biological Sciences, The University of Edinburgh, Edinburgh, United Kingdom; Duke University, UNITED STATES

## Abstract

In oocytes, where centrosomes are absent, the chromosomes direct the assembly of a bipolar spindle. Interactions between chromosomes and microtubules are essential for both spindle formation and chromosome segregation, but the nature and function of these interactions is not clear. We have examined oocytes lacking two kinetochore proteins, NDC80 and SPC105R, and a centromere-associated motor protein, CENP-E, to characterize the impact of kinetochore-microtubule attachments on spindle assembly and chromosome segregation in *Drosophila* oocytes. We found that the initiation of spindle assembly results from chromosome-microtubule interactions that are kinetochore-independent. Stabilization of the spindle, however, depends on both central spindle and kinetochore components. This stabilization coincides with changes in kinetochore-microtubule attachments and bi-orientation of homologs. We propose that the bi-orientation process begins with the kinetochores moving laterally along central spindle microtubules towards their minus ends. This movement depends on SPC105R, can occur in the absence of NDC80, and is antagonized by plus-end directed forces from the CENP-E motor. End-on kinetochore-microtubule attachments that depend on NDC80 are required to stabilize bi-orientation of homologs. A surprising finding was that SPC105R but not NDC80 is required for co-orientation of sister centromeres at meiosis I. Together, these results demonstrate that, in oocytes, kinetochore-dependent and -independent chromosome-microtubule attachments work together to promote the accurate segregation of chromosomes.

## Author Summary

In acentrosomal oocytes, spindle assembly depends on the chromosomes. The nature of the chromosome-microtubule interactions in oocytes that organize spindle bipolarity and orientation of the homologs has been unclear. We have found that several types of functional chromosome-microtubule interactions exist in oocytes, and that each type participates in unique aspects of chromosome orientation and spindle assembly. We present here a model for chromosome-based spindle assembly and chromosome movements in oocytes that highlights the multiple and unappreciated roles played by the kinetochores and has implications for how homologous chromosomes bi-orient during meiosis.

## Introduction

It is well established that oocyte spindle assembly in many organisms occurs in the absence of centrosomes [1–3]. Instead, chromatin-based mechanisms play an important role in spindle assembly. The interactions between chromosomes and microtubules are paramount in oocytes, necessary for both the assembly of the spindle and the forces required for chromosome segregation. Less well understood, however, is the nature of the functional connections between chromosomes and microtubules in these cells. The role of the kinetochores, the primary site of interaction between chromosomes and microtubules, is poorly understood in acentrosomal systems. For example, spindles will assemble and chromatin will move without kinetochores in both *Caenorhabditis elegans* and mouse oocytes [4, 5]. In addition, both *C*. *elegans* and mouse oocytes experience a prolonged period during which chromosomes have aligned but end-on kinetochore-microtubule attachments have not formed [6–8]. We have previously shown that the central spindle, composed of antiparallel microtubules that assemble adjacent to the chromosomes, is important for spindle bipolarity and homolog bi-orientation [9, 10]. These studies suggest that lateral interactions between the chromosomes and microtubules drive homolog bi-orientation, but whether these interactions are kinetochore-based is not clear.

There have been few studies directly analyzing kinetochore function in oocyte spindle assembly and chromosome segregation [5, 11, 12]. Assembling a functional spindle requires the initiation of microtubule accumulation around the chromatin, the organization of microtubules into a bipolar structure, and the maturation of the spindle from promoting chromosome alignment to promoting segregation. Whether the kinetochores are required for spindle assembly or the series of regulated and directed movements chromosomes undergo to ensure their proper partitioning into daughter cells is not known. In *Drosophila*, the chromosomes begin the process within a single compact structure called the karyosome [13]. Within the karyosome, centromeres are clustered prior to nuclear envelope breakdown (NEB) [14]. This arrangement, which is established early in prophase and maintained throughout diplotene/diakinesis, is also found in many other cell types [15]. It is possible that the function of centromere clustering is to influence the orientation of the centromeres on the spindle independent of chiasmata [16, 17]. Following NEB, the centromeres separate. In *Drosophila* oocytes, centromere separation depends on the chromosomal passenger complex (CPC) [10]. Whether this movement depends on interactions between chromosomes and microtubules remains to be established [10].

Following centromere separation, homologous centromeres move towards opposite spindle poles. During this time in *Drosophila* oocytes, the karyosome elongates and achiasmate chromosomes may approach the poles, separating from the main chromosome mass [18]. As prometaphase progresses, the chromosomes once again contract into a round karyosome. These chromosome movements appear analogous to the congression of chromosomes to the metaphase plate that ultimately results in the stable bi-orientation of chromosomes. In mitotic cells, congression depends on lateral interactions between kinetochores and microtubules [19, 20], and bi-orientation depends on the formation of end-on kinetochore-microtubule attachments [21, 22]. In oocytes, lateral chromosome-microtubule interactions have been suggested to be especially important, but how lateral and end-on kinetochore-microtubule attachments are coordinated to generate homolog bi-orientation has not been studied [9, 23].

To investigate the roles of lateral and end-on kinetochore-microtubule attachments in spindle assembly and prometaphase chromosome movements of acentrosomal oocytes, we characterized *Drosophila* oocytes lacking kinetochore components. The KNL1/Mis12/Ndc80 (KMN) complex is at the core of the kinetochore, providing a link between centromeric DNA and microtubules [24, 25]. Both KNL1 and NDC80 bind to microtubules *in vitro* [26], but NDC80 is required specifically for end-on kinetochore-microtubule attachments [24]. Therefore, we examined oocytes lacking either NDC80 to eliminate end-on attachments or the *Drosophila* homolog of KNL1, SPC105R, to eliminate all kinetochore-microtubule interactions. We also examined *Drosophila* oocytes lacking the centromere-associated kinesin motor CENP-E because CENP-E promotes the movement of chromosomes along lateral kinetochore-microtubule attachments in a variety of cell types [19, 20].

Our work has identified three distinct functions of kinetochores that lead to the correct orientation of homologs at meiosis I. First, SPC105R is required for the co-orientation of sister centromeres at meiosis I. This is a unique process that fuses sister centromeres, ensuring they attach to microtubules from the same pole at meiosis I. Second, lateral kinetochore-microtubule attachments are sufficient for prometaphase chromosome movements, which may be required for each pair of homologous centromeres to establish connections with microtubules from opposite poles. Third, end-on attachments are dispensable for prometaphase movement but are essential to stabilize homologous chromosome bi-orientation. Surprisingly, we found that although *Drosophila* oocytes do not undergo traditional congression of chromosomes to the metaphase plate, CENP-E is required to prevent chromosomes from becoming un-aligned and to promote the correct bi-orientation of homologous chromosomes. We also show that the initiation of acentrosomal chromatin-based spindle assembly does not depend on kinetochores, suggesting the presence of important additional interaction sites between chromosomes and microtubules. The stability of the oocyte spindle, however, becomes progressively more dependent on kinetochores as the spindle transitions from prometaphase to metaphase. Overall, this work shows that oocytes integrate several chromosome-microtubule connections to promote spindle formation and the different types of chromosome movements that ensure the proper segregation of homologous chromosomes during meiosis.

## Results

### Loss of SPC105R disrupts kinetochore assembly in *Drosophila* oocytes

To study the role of kinetochores in oocyte spindle assembly and chromosome orientation, we sought to eliminate kinetochore function in *Drosophila* oocytes. Mutations in *Drosophila* kinetochore genes are lethal prior to the initiation of oogenesis [27–30], and germline clones of kinetochore mutants failed to complete oogenesis (S1 Table). Therefore, we used RNAi to deplete kinetochore proteins in *Drosophila* oocytes (see Materials and Methods). Mitotic cells lacking NDC80 have persistent lateral kinetochore-microtubule attachments [31], while loss of SPC105R (the *Drosophila* homolog of KNL1) results in destabilization of all kinetochore-microtubule attachments [32]. Therefore, we decided to use *Ndc80* and *Spc105R* depletion to examine the roles of kinetochores and discriminate between the roles of lateral and end-on kinetochore-microtubule attachments in oocytes. One *Ndc80* (GL00625) and two *Spc105R* (GL00392 and HMS01548) RNAi constructs were obtained. In oocytes, expression of these constructs knocked down *Ndc80* gene expression by 94% and *Spc105R* gene expression by 87% and 96%, respectively. No significant phenotypic differences were observed between the two *Spc105R* constructs; therefore, for simplicity all experiments shown used only the GL00392 hairpin except where noted.

**Fig 1:**
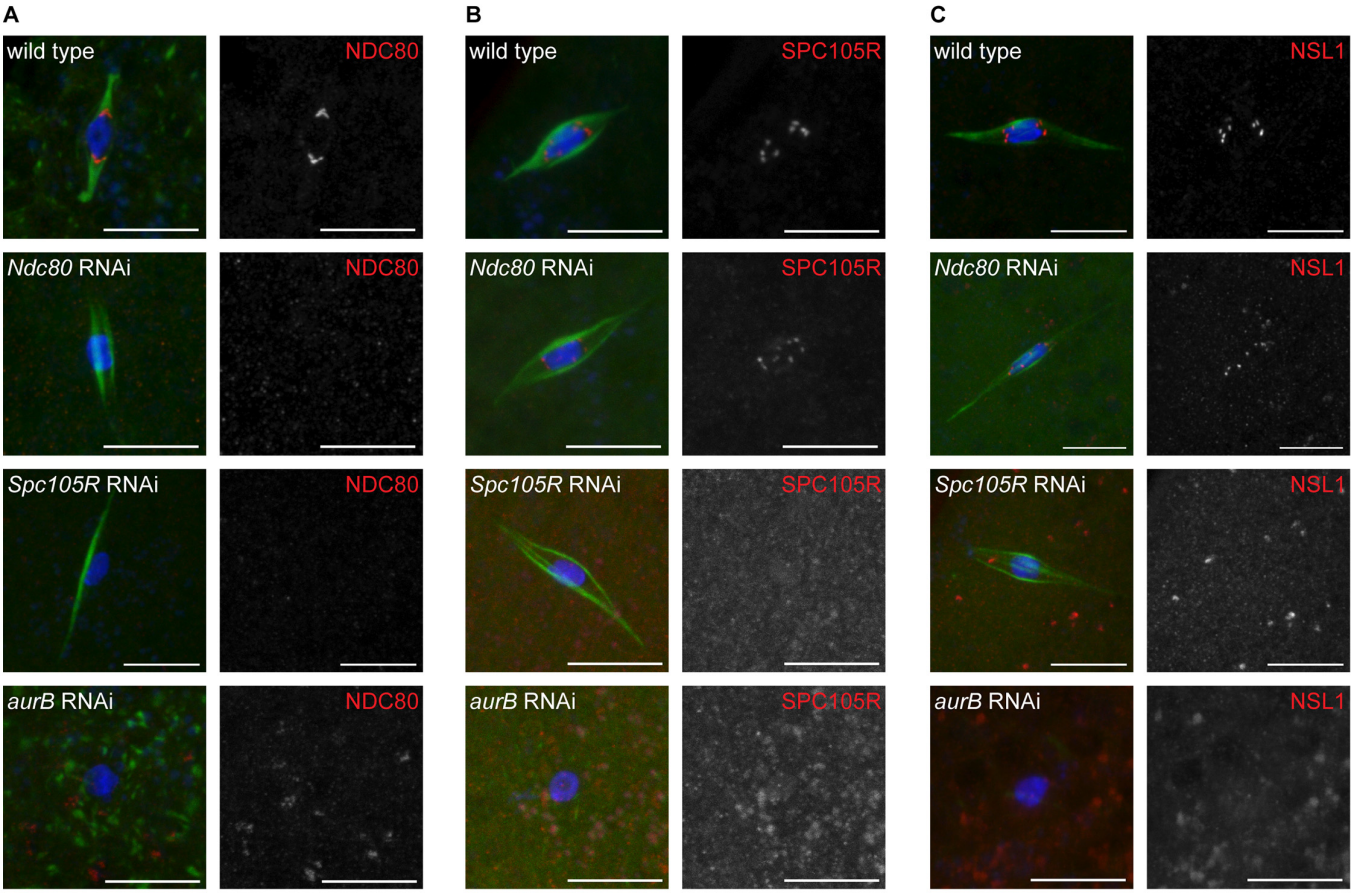
Loss of SPC105R or the CPC disrupts kinetochore assembly in oocytes. Confocal images of localization of (A) NDC80, (B) SPC105R, and (C) NSL1 (Mis12 complex) in wild-type oocytes and after knockdown of *Ndc80*, *Spc105R*, or *aurB*. DNA is shown in blue and tubulin is shown in green in merged images. Kinetochore components are shown in red in merged images and white in single channel images. Scale bars represent 10 μm.

We found that localization of NDC80 and SPC105R to kinetochores was absent in *Ndc80*- or *Spc105R*-depleted oocytes, respectively (S2 Table and Fig 1A and 1B), showing that the RNAi knockdown was effective. In addition, NDC80 and NSL1 (a member of the Mis12 complex) failed to localize to kinetochores in *Spc105R*-depleted oocytes (S2 Table and Fig 1A and 1C), while both SPC105R and NSL1 localized to kinetochores in *Ndc80*-depleted oocytes (S2 Table and Fig 1B and 1C). These results are consistent with results from mitotic cells in *Drosophila* embryos and cell culture [28, 33]: localization of KMN complex proteins in *Drosophila* depends on SPC105R but not NDC80.

### Loss of NDC80 or SPC105R disrupts kinetochore-microtubule attachments in oocytes

**Fig 2:**
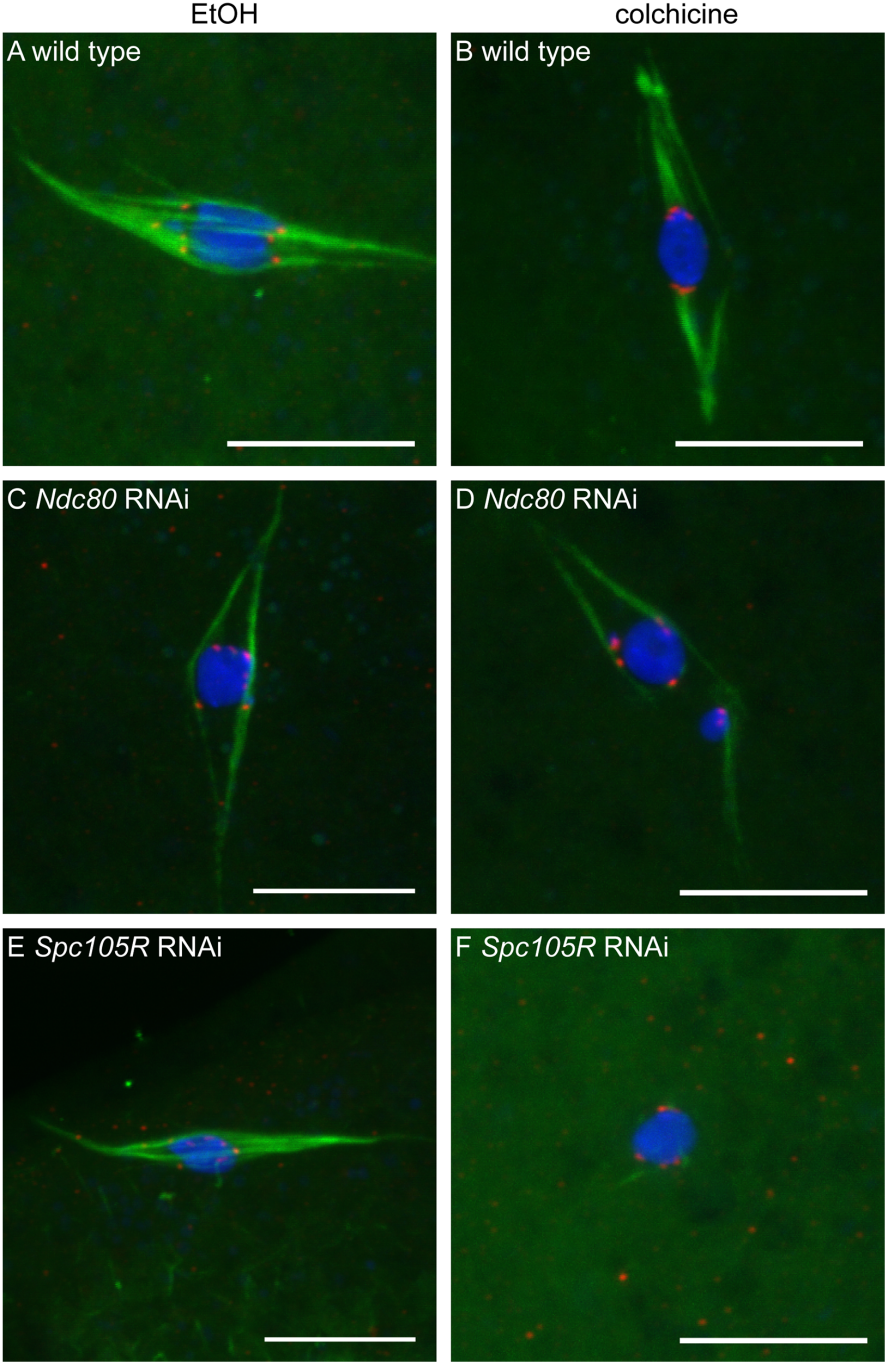
Loss of NDC80 or SPC105R disrupts interactions between kinetochores and microtubules in oocytes. Confocal images of wild-type oocytes (A,B) and after knockdown of *Ndc80* (C,D) or *Spc105R* (E,F). Oocytes were treated with either ethanol (EtOH) (A,C,E) or colchicine (B,D,F). DNA is shown in blue, tubulin is shown in green, and CENP-C is in red. Scale bars represent 10 μm

To determine if kinetochore-microtubule attachments are affected in oocytes depleted of kinetochore components, we examined microtubule localization relative to the centromere protein, CENP-C. The robust central spindle makes it difficult to directly observe kinetochore microtubules; therefore, we used conditions that depolymerize central spindle microtubules to directly observe kinetochore microtubules. Wild-type oocytes exposed to colchicine (see Materials and Methods for details) resulted in the loss of most spindle microtubules except for those that ended at the centromeres (Fig 2B, 17/18 oocytes). In contrast, in colchicine-treated oocytes lacking NDC80, the microtubules were weaker, and those remaining often appeared to be interacting laterally with the centromeres (Fig 2D, 7/12 oocytes). In colchicine-treated oocytes lacking SPC105R, no end-on kinetochore-microtubule attachments were observed (0/12 oocytes), and we observed some oocytes in which all of the microtubules were eliminated (Fig 2F, 3/12 oocytes). These results suggest that NCD80 is required for end-on kinetochore-microtubule attachments, while all kinetochore-microtubule interactions depend on SCP105R.

**Fig 3:**
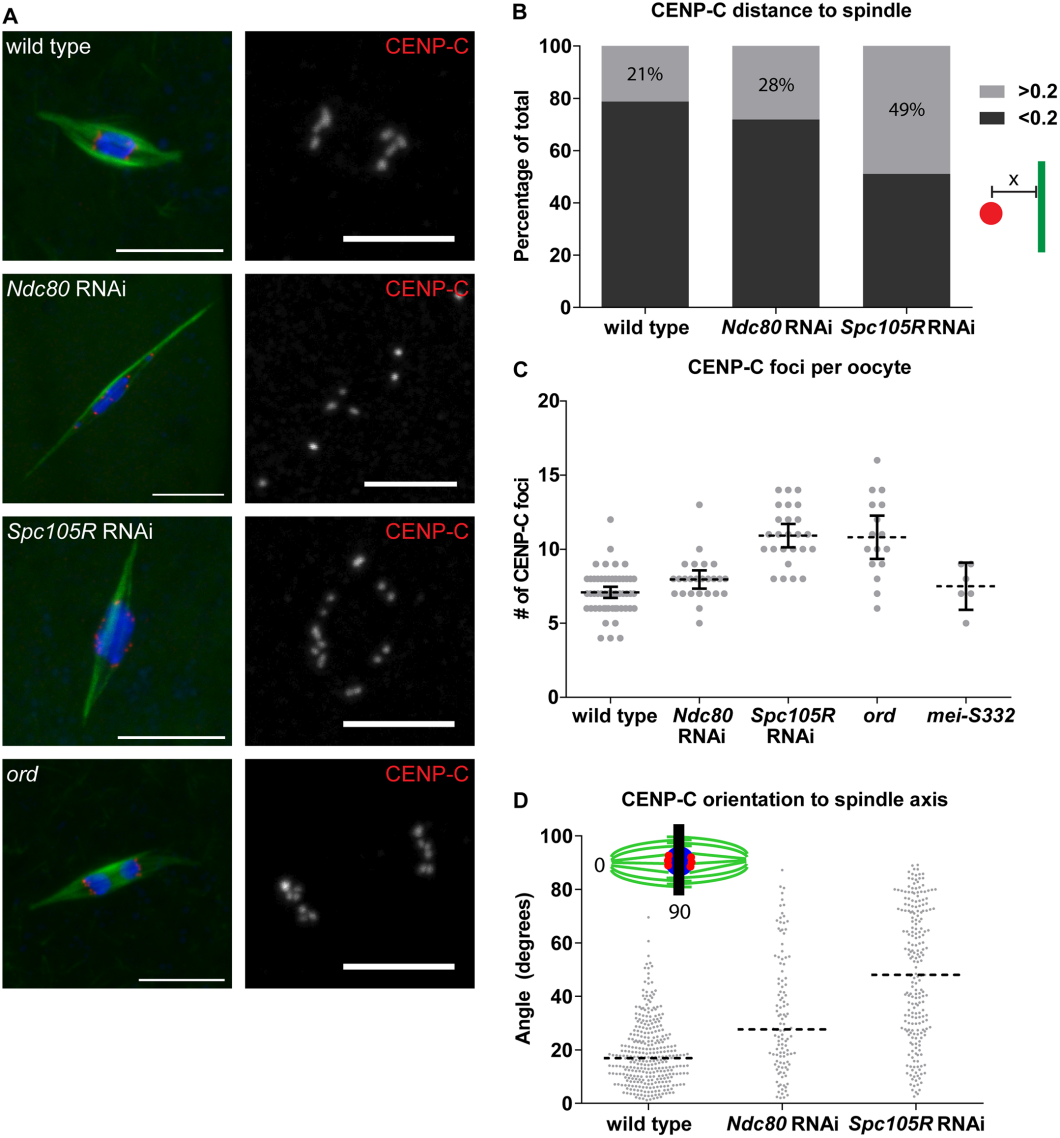
Chromosome orientation depends on the kinetochore in oocytes. (A) Confocal images of the centromere protein CENP-C (red in merged images, white in single channel) in wild-type oocytes, after knockdown of *Ndc80* or *Spc105R*, and in *ord* mutants. Single channel images are zoomed in relative to merged to highlight CENP-C foci. In *ord* mutants, due to defects in cohesion or crossing over, precocious anaphase is observed [80, 81]. DNA is in blue and tubulin is in green in merged images. Scale bars represent 10 μm in merged, 5 μm in CENP-C single channel images. (B) Bar graph showing the ratio of CENP-C foci closer to (dark gray) and further from (light gray) the spindle than 0.2 μM. Inset shows a centromere in red, a microtubule in green, and the distance measurement denoted by “x”. (C) Dot plot of the number of CENP-C foci per oocyte in wild type, after *Ndc80* or *Spc105R* knockdown, in *ord* mutants, and in *mei-S332* mutants (encoding the *Drosophila* homolog of Shugoshin). Horizontal dotted lines show the mean, error bars show 95% confidence intervals. (D) Dot plot of the angle of CENP-C foci with respect to the spindle axis in wild-type oocytes and after knockdown of *Ndc80* or *Spc105R*. Horizontal dotted lines show the median. The red bar in the inset shows a line perpendicular to the spindle axis: centromeres located on this line would result in 90 degree angle measurements, while centromeres in line with the spindle axis measure 0 degrees. Differences in the width of the karyosome, in wild-type oocytes (3.13 μm), and after knockdown of *Ndc80* (3.43 μm) or *Spc105R* (3.32 μm), are not great enough to explain the differences in angles of CENP-C foci.

To confirm that SPC105R, but not NDC80, is required for kinetochore-microtubule interactions, we measured the distance between each centromere and the nearest microtubules in oocytes not treated with colchicine. In wild type, the majority of centromeres were within 0.2 μm of the microtubules (Fig 3A and 3B). A similar frequency was found with loss of NDC80, suggesting these defective kinetochores still interacted with the microtubules (Fig 3A and 3B, *P* = 0.07). Oocytes lacking SPC105R, however, had significantly fewer centromeres within 0.2 μm of the microtubules (Fig 3A and 3B, *P* = <0.0001). Based on the results with colchicine, it is likely that centromeres lacking SPC105R move within 0.2 μm of a microtubule by chance. These results suggest SPC105R, but not NDC80, is required for the kinetochores to attach to the microtubules.

To examine microtubule interactions using a functional readout for end-on attachments, we examined the localization of ROD. ROD is part of the RZZ complex, which localizes to kinetochores until the formation of end-on kinetochore-microtubule attachments when it leaves the kinetochore by streaming along the kinetochore microtubules [34]. In wild-type oocytes, ROD was present at kinetochores, and we observed streams of ROD along microtubules (S1 and S2 Figs), suggesting that end-on kinetochore-microtubule attachments indeed form in *Drosophila* oocytes. In *C*. *elegans*, localization of the RZZ complex to the kinetochore depends on KNL-1, the homolog of *Drosophila* SPC105R [35]. Similarly, we did not observe localization of ROD to kinetochores in *Spc105R*-depleted oocytes (S1 Fig). In *Ndc80*-depleted oocytes, however, ROD was present at kinetochores, but in most oocytes we did not observe streaming along the microtubules (S1 Fig). Therefore, the lack of ROD streaming demonstrates that end-on kinetochore-microtubule attachments do not form in the absence of NDC80.

### Lateral kinetochore-microtubule attachments drive prometaphase chromosome movements

To determine the role of kinetochore-microtubule attachments in oocyte chromosome movement, we examined prometaphase karyosome configurations after knockdown of *Ndc80* or *Spc105R*. During prometaphase I, chromosomes undergo movements to facilitate contact with the spindle, a process that may be required for chromosome alignment [18]. In *Drosophila* oocytes, chromosomes are compacted into a karyosome [13] so congression to the metaphase plate is unnecessary. Prometaphase chromosome movements still occur and are visible through the elongation of the karyosome and the separation of achiasmate chromosomes from the karyosome (S3 Fig). Collections of oocytes can be enriched for either prometaphase or metaphase depending on how the females are treated (see Materials and Methods) [18]. We found that prometaphase karyosome configurations in *Ndc80*-depleted oocytes were similar in frequency to wild type (28% vs. 26%; Table 1). In contrast, in *Spc105R*-depleted oocytes, elongation of the karyosome and separation of achiasmate chromosomes was rarely observed (6% of oocytes; Table 1). Therefore, prometaphase chromosome movements depend on SPC105R, but not NDC80, suggesting that lateral kinetochore-microtubule attachments are sufficient to drive prometaphase chromosome movements in oocytes.

**Table 1:** Prometaphase karyosome configurations in the absence of kinetochore components.

	hairpin	round	prometaphase^a^	split^b^	n	*P*
wild type	-	121 (71%)	45 (26%)	5 (3%)	171	NA
*Ndc80* RNAi	GL00625	47 (66%)	20 (28%)	4 (6%)	71	0.8^c^
*Spc105R* RNAi	GL00392	75 (87%)	5 (6%)	6 (7%)	86	<0.0001^c^
*Spc105R* RNAi	HMS01548	52 (83%)	4 (6%)	7 (11%)	63	0.0005^c^
*cmet* RNAi	GL00404	66 (70%)	14 (15%)	14 (15%)	94	0.0007^d^
*Cenp-E* ^*141*^	-	25 (51%)	3 (6%)	21 (43%)	49	<0.0001^d^
*Ndc80 cmet* RNAi	GL00625, GL00404	25 (50%)	5 (10%)	20 (40%)	50	<0.0001^d^
*Spc105R cmet* RNAi	HMS01548, GL00404	47 (94%)	1 (2%)	2 (4%)	50	0.7^d^

^a^ Prometaphase defined as karyosome in a figure eight shape and/or 4th chromosomes separated from main karyosome mass. See S3 Fig.

^b^ Karyosome is separated into two or more masses of chromosomes

^c^ Fisher's exact test comparing prometaphase/non-prometaphase to wild type

^d^ Fisher’s exact test comparing split/non-split to wild type

### Chromosome co-orientation at meiosis I depends on SPC105R

Karyosome morphology does not reveal information about individual chromosomes. Therefore, to gain a more direct picture of chromosome behavior in the absence of kinetochore proteins, we examined the position of all centromeres using immunolocalization of CENP-C (Fig 3A). *Drosophila* have four pairs of homologous chromosomes. Because each homologous chromosome is formed from four chromatids, 16 centromeres are present at meiosis I. During meiosis I, however, sister centromeres are fused to promote their co-orientation toward one pole. In agreement with this, we found an average of 7.0 CENP-C foci were visible per wild-type oocyte (Fig 3C). The deviation from the expected value of eight is due to an inability to resolve centromeres of different chromosomes that are fortuitously close together. In *Spc105R*-depleted oocytes, the average CENP-C foci number was significantly elevated to 11.1 (*P* = <0.0001; Fig 3C), suggesting a loss of co-orientation. This difference is in contrast to *Ndc80*-depleted oocytes that had an average of 7.7 CENP-C foci, which does not differ significantly from wild type (*P* = 0.13; Fig 3C). One possibility is that kinetochore-microtubule attachments are required for co-orientation. An alternative, however, is that SPC105R is required for the kinetochore localization of proteins that do not depend on NDC80 [36]. In yeast, the monopolin complex promotes co-orientation [37], and MEIKIN provides this function in vertebrates [38]. Perhaps SPC105R is required for the kinetochore localization of the as-yet-unidentified invertebrate functional equivalent of monopolin/MEIKIN.

Although little is known about the molecular mechanism of co-orientation in metazoan oocytes, sister chromatid cohesion has been shown to be involved [22]. To determine whether loss of cohesion results in sister centromere separation in *Drosophila* oocytes, we examined *ord* mutants (Fig 3A and 3C). ORD is required for sister chromatid cohesion during meiosis [39]. In *ord* mutants, we observed an average of 10.8 CENP-C foci, which is not significantly different from *Spc105R*-depleted oocytes (*P* = 0.76; Fig 3C). We also examined sister centromere separation in *mei-S332* mutants, which mutate the *Drosophila* homolog of Shugoshin [40]. With an average of 7.5 CENP-C foci, this is not significantly different than wild type (*P* = 0.47; Fig 3C), but is significantly different from both *Spc105R* depletion (*P* = 0.0005) and *ord* mutants (*P* = 0.01), consistent with the conclusion that MEI-S332 function is not required until anaphase I [22]. These results suggest that SPC105R is required for co-orientation in oocytes, perhaps through the protection of sister chromatid cohesion during meiosis I.

### Lateral and end-on kinetochore-microtubule attachments are required for orientation towards a spindle pole

While only *Spc105R*-depleted oocytes had a co-orientation defect, immunolocalization of CENP-C revealed a defect present in both *Ndc80*- and *Spc105R*-depleted oocytes. In wild-type *Drosophila* oocytes, centromere foci are clustered into two groups at the edge of the karyosome closest to each spindle pole (Fig 3A). This represents when microtubule connections to the spindle poles pull homologous chromosomes in opposite directions. In *Ndc80*- and *Spc105R*-depleted oocytes, the centromere foci were not clustered into two groups oriented toward each spindle pole, but rather were scattered around the karyosome (Fig 3A). This is a failure of the centromeres to orient towards a spindle pole.

To quantify this phenotype, we measured the angle of displacement of each CENP-C focus with respect to the axis of the half spindle, defined by the line between a point at the spindle pole and the center point of the karyosome (Fig 3D). Oriented centromeres have measurements as low as 0 degrees, while centromeres that fail to orient and are scattered around the karyosome result in angle measurements up to 90 degrees. In wild type, CENP-C foci angles had a median value of 17 degrees (Fig 3D). CENP-C angles in *Ndc80*- or *Spc105R*-depleted oocytes were skewed significantly higher with median values of 28 degrees and 48 degrees, respectively (*P* = <0.0001 for each; Fig 3D), demonstrating that kinetochore-microtubule attachments are required for chromosomes to orient towards a spindle pole. However, there was also a significant difference in CENP-C foci angles between *Ndc80*- and *Spc105R*-depleted oocytes (*P* = <0.0001). This is reflected in the data by the greater number of oocytes with angles close to 90 degrees in *Spc105R*-depleted oocytes. These data show that loss of NDC80 disturbs chromosome orientation, although not as dramatically as loss of SPC105R. To explain this difference, we suggest that lateral kinetochore-microtubule attachments are sufficient for some partial or unstable chromosome orientation, while end-on attachments cement orientation towards a spindle pole.

### Bi-orientation of homologous centromeres depends on lateral kinetochore-microtubule attachments

**Fig 4:**
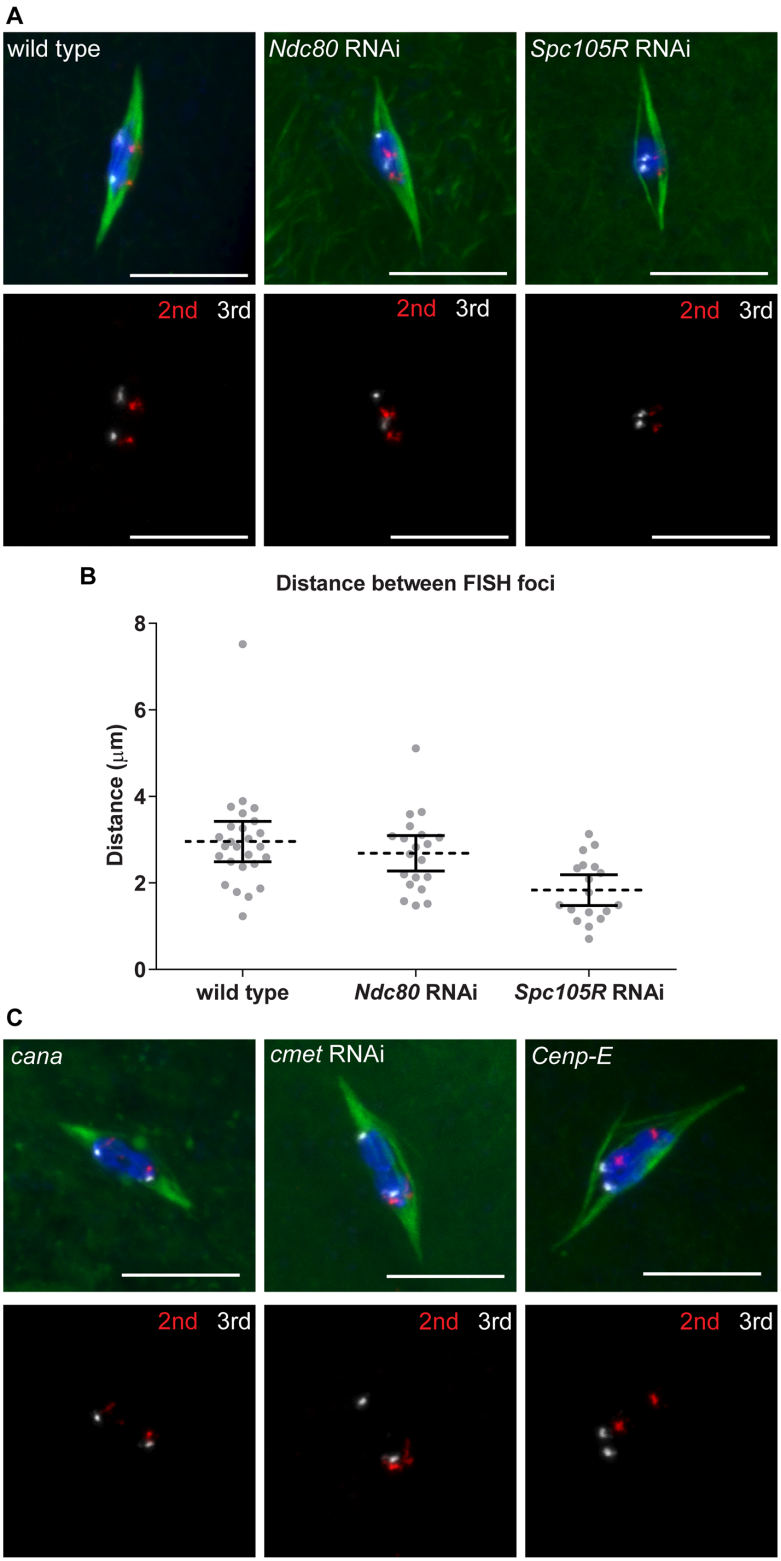
Homologous chromosome bi-orientation depends on kinetochores and CENP-E in oocytes. Confocal images of FISH probes marking the 2^nd^ (red) and 3^rd^ (white) chromosome centromeres. DNA is in blue and tubulin is in green in merged images. Only FISH probes are shown in the panel below each merged image. Scale bars represent 10 μm. (A) Oocytes from wild type and after knockdown of *Ndc80* or *Spc105R*. (B) Dot plot of the distance between pairs of FISH foci in wild-type oocytes and after knockdown of *Ndc80* or *Spc105R*. Horizontal dotted lines show the mean, error bars show 95% confidence intervals. (C) Oocytes from *cana/Df*, after knockdown of *cmet*, and *Cenp-E* germline clones. The data is summarized in Table 2.

Karyosome morphology and immunolocalization of CENP-C in *Ndc80*- or *Spc105R*-depleted oocytes suggested kinetochore-microtubule attachments allowed chromosomes to orient towards a spindle pole. To test whether each chromosome associated randomly with a pole, or if homologs oriented towards opposite poles (“bi-orientation”), we used FISH to examine specific chromosomes. Chromosome bi-orientation at meiosis I depends on the establishment of connections between homologous chromosome pairs and opposite spindle poles. When this occurs, tension across the homologous chromosome pair generates an increase in the inter-homolog centromere distance. We used FISH probes to the repetitive sequences present at the centromeres of the second and third chromosomes to determine directly whether the separation of homologous centromeres away from each other depends on kinetochores and their end-on attachment to microtubules in oocytes (Fig 4A). In wild type, we observed an average distance between homologous centromeres of 3.0 μm (Fig 4B). In *Ndc80*-depleted oocytes, the average distance between homologous centromeres was not significantly reduced (2.7 μm, *P* = 0.4; Fig 4B), suggesting that end-on kinetochore-microtubule attachments are not required for homologous centromeres to move away from each other. In contrast, in *Spc105R*-depleted oocytes, the average distance was significantly reduced to 1.8 μm (*P* = 0.0007; Fig 4B). These results suggest that lateral kinetochore-microtubule attachments are sufficient for homologous centromeres to orient towards a spindle pole and separate from each other in what may be the first step in the bi-orientation process.

### CENP-E is required for chromosome alignment and bi-orientation

Because CENP-E is a kinesin motor involved in the lateral movement of chromosomes along microtubules, we hypothesized that CENP-E could mediate some of the kinetochore-dependent movements that depend on SPC105R but not NDC80. *Drosophila melanogaster* is unusual because it has two *Cenp-E* genes, *cana* and *cmet*, arranged in inverse orientation on the chromosome (S4 Fig). The proteins encoded by *cana* and *cmet* show considerable sequence similarity throughout their motor and stalk domains (42% identical overall), and only 6 out of the 12 sequenced *Drosophila* species have two copies of *Cenp-E*, suggesting a recent duplication event.

It was previously shown that *cmet* mutants are inviable [41]. We generated *cana* mutants and *cana cmet* double mutants, which we refer to as *Cenp-E* mutants (see Materials and Methods for details). Like *cmet* mutants, *Cenp-E* mutants are inviable; however, *cana* mutants are viable and fertile. To determine the function of CENP-E in chromosome movement in oocytes, we focused on *cana* hemizygous mutants, depletion of *cmet* by RNAi (GL00404 from TRiP), and *Cenp-E* germline clones generated using the dominant female sterile technique [42]. In oocytes, expression of the GL00404 construct knocked down *cmet* gene expression by 75%.

**Fig 5:**
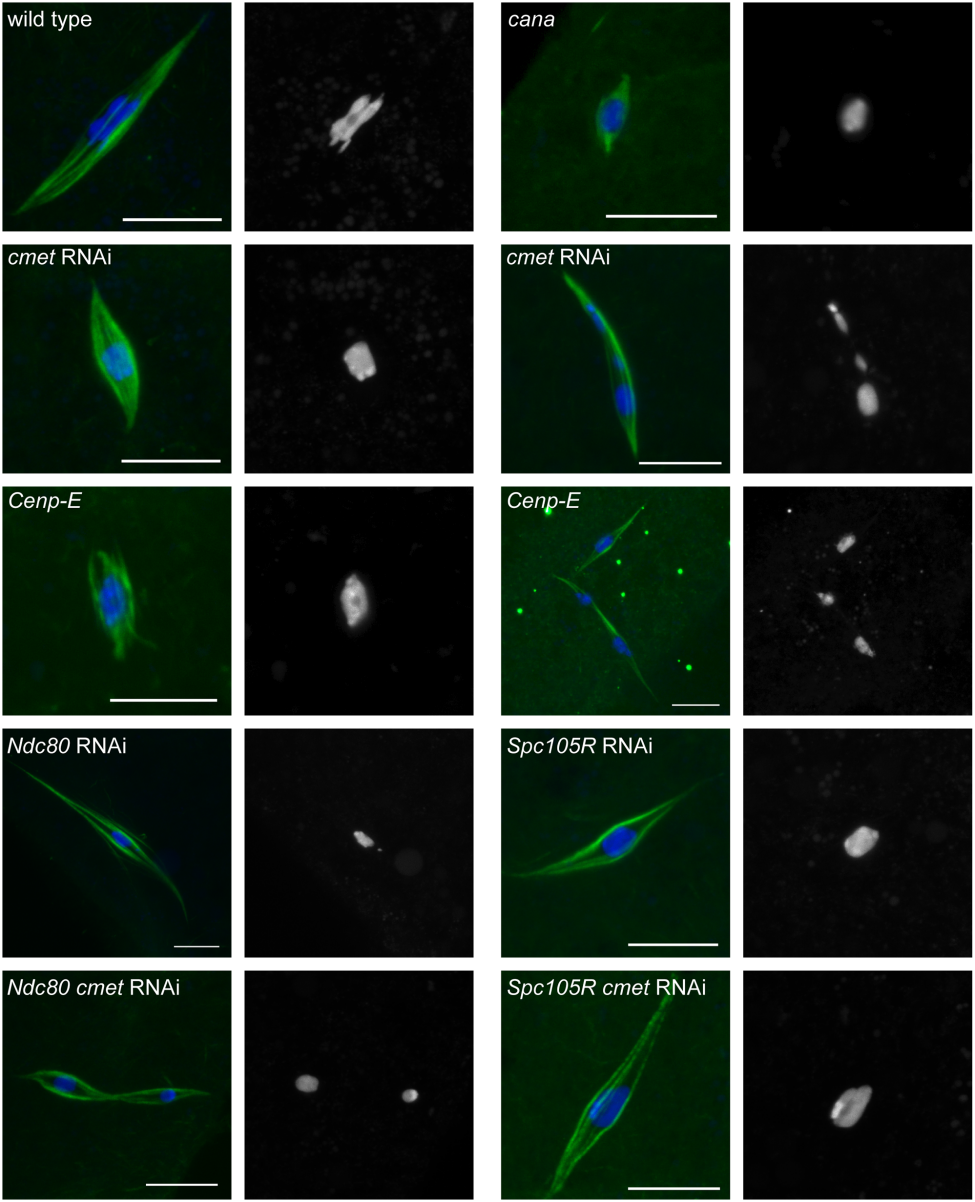
Loss of CENP-E disrupts chromosome alignment in oocytes. Confocal images of karyosome and spindle organization in oocytes from wild type, *cana/Df*, after knockdown of *cmet*, *Cenp-E* germline clones, and after knockdown of *Ndc80*, *Spc105R* (HMS01548), *Ndc80* and *cmet*, or *Spc105R* (HMS01548) and *cmet*. DNA is in blue and tubulin is in green in merged images. Single channel images show DNA in white. Scale bars represent 10 μm.

In *cana* mutant, *cmet*-depleted, or *Cenp-E* mutant oocytes, bipolar spindles formed (Fig 5). However, in *cmet*-depleted or *Cenp-E* mutant oocytes, the karyosome frequently split into multiple masses (Fig 5 and Table 1 and S3 Table). This karyosome defect was more frequent in *Cenp-E* mutant oocytes than in *cmet*-depleted oocytes (43% vs 15%, *P* = 0.001), demonstrating that both CENP-E homologs are required for proper karyosome organization. CANA and CMET are partially redundant because CMET is necessary for karyosome organization even when there is a functional copy of CANA. These results are the first evidence that the second *Drosophila* CENP-E homolog CANA is functional. Additionally, these results suggest that, although traditional chromosome congression does not occur in *Drosophila* oocytes, CENP-E is required to prevent chromosomes from becoming un-aligned and separated from the main karyosome mass.

To further explore the role of CENP-E in chromosome movements in oocytes, we examined the orientation of centromeres using FISH. Because *cana* mutants are fertile and do not exhibit chromosome segregation errors, not surprisingly we found no defect in centromere orientation (Fig 4C and Table 2). In contrast, homologous centromeres were frequently mis-oriented in both *cmet*-depleted and *Cenp-E* mutant oocytes (Fig 4C and Table 2). The frequency of mis-orientation was similar between *cmet*-depleted and *Cenp-E* mutant oocytes (29% vs 24%, *P* = 0.7), suggesting that CMET is the primary CENP-E homolog functioning in bi-orientation. Importantly, the *cmet* mis-orientation phenotype is distinct from either *Ndc80* or *Spc105R* depletion in that centromeres are not scattered around the karyosome: they orient, but often towards the wrong spindle pole (Fig 4C). This suggests that stable end-on kinetochore-microtubule attachments are not eliminated in the absence of CENP-E, but that CENP-E is required for establishing the correct kinetochore-microtubule attachments to direct homologs toward opposite spindle poles.

**Table 2:** Centromere bi-orientation in the absence of CANA and CMET.

	bi-oriented	mono-oriented	n	*P* ^a^	other^b^
wild type	106 (95%)	5 (5%)	111	NA	19
*cana/Df*	17 (94%)	1 (6%)	18	1.0	0
*cmet* RNAi	65 (71%)	27 (29%)	92	<0.0001	23
*Cenp-E*	25 (76%)	8 (24%)	33	0.002	17

^a^ Fisher’s exact test comparing bi/mono-orientation to wild type

^b^ Oocytes in which orientation could not be determined because spindle poles were poorly defined, centromeres were not pulled towards a pole, and/or the chromosomes were dispersed.

To determine more directly the nature of microtubule attachments in the absence of CENP-E, we examined the localization of ROD. ROD accumulates at kinetochores until the formation of end-on kinetochore-microtubule attachments [34]. We found that ROD was present at kinetochores and streaming along microtubules in *cmet*-depleted oocytes, similar to wild type (S1 Fig). This demonstrates that CENP-E is not required to form end-on kinetochore-microtubule attachments. However, one known role of CENP-E is in the regulation of end-on kinetochore-microtubule attachment stability [43, 44]. Consistent with this, upon closer investigation using live imaging, we observed an increased frequency of ROD at the kinetochores in *cmet*-depleted oocytes (S2 Fig). Kinetochores that are not streaming ROD should undergo re-orientation to achieve stable bi-orientation. This can be observed in live imaging of wild-type oocytes because kinetochores with accumulated ROD change position within the karyosome (S5 Fig). The kinetochores that accumulated ROD in the absence of CMET, however, often failed to change position within the karyosome (S5 Fig), suggesting that in the absence of CMET, the ability to re-orient following a failure to bi-orient is defective.

### CENP-E prevents chromosome movement via lateral kinetochore-microtubule attachments

Because the karyosome is maintained in the absence of the kinetochore components NDC80 or SPC105R (Table 1), active congression via kinetochore-microtubule attachments may not be required for chromosome organization in *Drosophila* oocytes. On the other hand, the karyosome splits apart in the absence of CENP-E, resulting in the un-alignment of chromosomes (Table 1). Because CENP-E is typically thought to move chromosomes via lateral kinetochore-microtubule attachments, we wanted to test whether the splitting apart of the karyosome in the absence of CENP-E depends on kinetochore-microtubule attachments.

We examined karyosome configurations in two types of oocytes: those depleted of both *cmet* and *Ndc80* or both *cmet* and *Spc105R* (Fig 5 and Table 1). We found that loss of CMET in the absence of SPC105R did not result in karyosome splitting (Table 1). This suggests that the movement of chromosomes that results in splitting of the karyosome depends on kinetochore-microtubule attachments. On the other hand, the karyosome split apart in *Ndc80 cmet* double-depleted oocytes (Table 1), suggesting that lateral kinetochore-microtubule attachments are sufficient for the splitting of the karyosome, and that CMET opposes this movement. Strikingly, the karyosome defect was enhanced in *Ndc80 cmet* double-depleted oocytes compared to *cmet*-depleted oocytes (40% vs. 15%, *P* = 0.004). In fact, *Ndc80 cmet* double-depleted oocytes are not significantly different from *Cenp-E* oocytes (40% vs. 43%, *P* = 0.8). One possibility is that NDC80 is required for CANA function such that loss of NDC80 and CMET together effectively recapitulates the complete loss of CENP-E. Alternatively, NDC80 (via end-on kinetochore-microtubule attachments) and CMET (via lateral kinetochore-microtubule attachments) may work together to oppose the forces driving chromosome un-alignment. In any case, these results demonstrate that CENP-E prevents chromosome un-alignment via lateral-kinetochore microtubule attachments.

### Oocyte spindle stability depends on kinetochores and the central spindle

**Fig 6:**
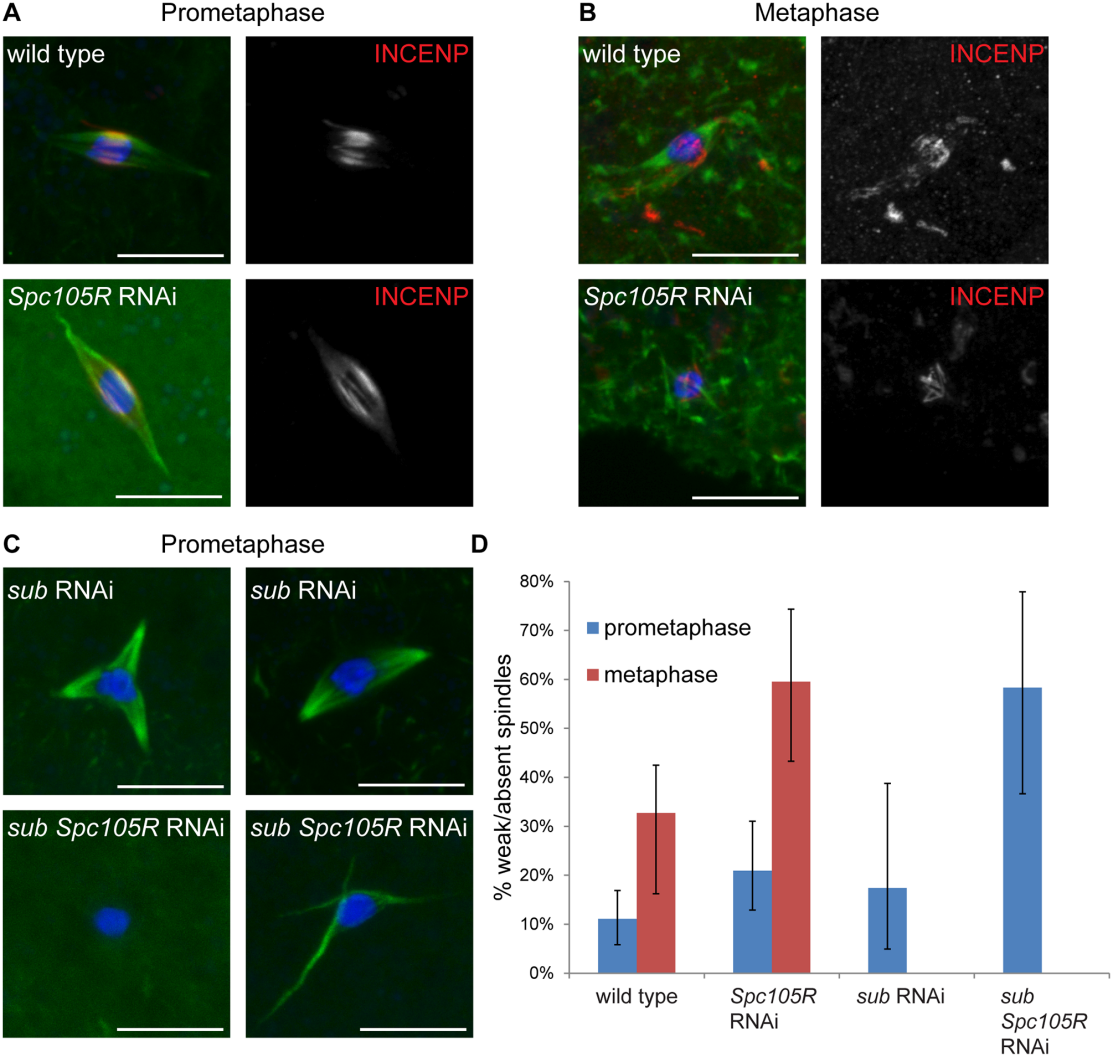
Prometaphase spindle stability depends on both kinetochore and central spindle components in oocytes. In all images, DNA is in blue, tubulin is in green, and the scale bars represent 10 μm. (A,B) Confocal images of wild-type oocytes and after *Spc105R* knockdown from prometaphase-enriched (A) and metaphase-enriched (B) collections. The CPC component INCENP is in red in merged images, white in single channel images. (C) Confocal images of *sub*-depleted and *sub Spc105R* double-depleted oocytes. For *sub*-depleted oocytes, a tripolar (left) and bipolar (right) spindle are shown. Monopolar spindles were also observed [9, 47]. In all *sub*-depleted oocytes, the prominent central spindle is missing. For *sub Spc105R* double-depleted oocytes, the absence of a spindle (left) and a spindle with thin and disorganized microtubules (right) are shown. (D) Graph showing the percentage of weak/absent spindles during prometaphase in wild-type oocytes (n = 171) and after *Spc105R* (n = 86), *sub* (n = 23), or *sub Spc105R* (n = 24) depletion, and metaphase in wild-type oocytes (n = 110) and after *Spc105R* depletion (n = 42). The frequency of weak/absent spindles at metaphase in *sub*- and *sub Spc105R*-depleted oocytes was not determined. Error bars show 95% confidence intervals.

Our results thus far show that kinetochores participate in chromosome alignment, bi-orientation, and co-orientation in *Drosophila* oocytes. Since chromatin-mediated pathways direct spindle assembly in oocytes [45], we investigated the contribution of the kinetochores to spindle assembly and stability at prometaphase I and metaphase I. Metaphase I-arrested spindles tend to be shorter than prometaphase spindles with less prominent central spindles (Fig 6A and 6B). In fact, we observed that spindles were weak, that is very small, faint, or lacking microtubules entirely (indicated below as “weak/absent”), more frequently in metaphase-enriched oocyte samples (33%, *P* = <0.0001) (see Materials and Methods for details of how metaphase- or prometaphase-enriched samples are collected) than in prometaphase-enriched oocyte samples (11%) (Fig 6D). This difference suggests that in *Drosophila* oocytes, spindle assembly proceeds via an elongation phase during which spindles are robust (prometaphase), followed by a contraction phase in which microtubule density decreases (metaphase).

We found that *Spc105R*-depleted oocytes form bipolar spindles in prometaphase-enriched oocyte collections. In prometaphase-enriched collections, weak/absent spindles were increased in *Spc105R*-depleted oocytes compared to wild type (21%, *P* = 0.04; Fig 6D). This difference suggests that the prometaphase spindle is destabilized in the absence of kinetochore components. Weak/absent spindles were significantly increased compared to wild type in metaphase-enriched collections of *Spc105R*-depleted oocytes (60%, *P* = 0.003; Fig 6B and 6D). These results suggest that kinetochore microtubules contribute early to the organization of the prometaphase spindle, and then form the majority of microtubules in the metaphase-arrested spindle.

These results make predictions about the microtubules that assemble in *Spc105R*-depleted oocytes. First, although these spindles are bipolar, they should lack kinetochore microtubules. Indeed, *Spc105R*-depleted oocyte spindles appear hollow, as if they are missing the microtubules that, in wild type, end at the chromosomes (Figs 1, 3A, 4A, 5 and 6A). *Ndc80*-depleted oocytes also form hollow spindles (Figs 1, 3A, 4A and 5); therefore, the stable kinetochore microtubules are most likely only those that form end-on attachments. The microtubules in these hollow spindles could depend on the prominent central spindle that forms in *Drosophila* oocytes and is required for bipolarity and chromosome bi-orientation [10, 46]. To directly determine whether the central spindle forms properly in the absence of kinetochores, we examined localization of INCENP, a member of the CPC, in *Spc105R*-depleted oocytes. We found that INCENP localized normally in the hollow spindles from prometaphase-enriched collections (Fig 6A). Indeed, most spindles in *Spc105R*-depleted prometaphase oocytes are bipolar, suggesting the central spindle is sufficient to organize the spindle poles. However, the central spindle was disorganized in metaphase-enriched *Spc105R*-depleted oocytes (Fig 6B), indicating that kinetochores contribute to the stability of the central spindle at metaphase.

If all microtubules present in *Spc105R*-depleted oocytes are associated with the central spindle, then the meiotic spindle is likely composed of two types of microtubules: kinetochore-dependent and central spindle-dependent. To test this hypothesis, we knocked down both *subito*, which is required for the formation of the central spindle [46], and *Spc105R* in oocytes. We found that *sub*-depleted oocytes had polarity defects similar to *sub* null mutants [46, 47] (Fig 6C), but this depletion did not significantly increase weak/absent spindles in prometaphase-enriched collections (17%, *P* = 0.5; Fig 6D). In contrast, *sub Spc105R* double depletion resulted in a significant increase in weak/absent spindles in prometaphase-enriched collections (58%, *P* = <0.0001), comparable to the spindle destabilization observed in metaphase-enriched collections from *Spc105R*-depleted oocytes (Fig 6C and 6D). These results suggest that both the organization and the stability of the prometaphase oocyte spindle depend on kinetochores and the central spindle. The metaphase-arrested spindle, on the other hand, depends mostly on kinetochore microtubules.

### Kinetochore assembly depends on the CPC

The CPC is required for the assembly of all microtubules around the karyosome in *Drosophila* oocytes (Fig 1) [10]. The CPC is also required for localization of central spindle components such as SUB [10]. Because we have shown that kinetochores and the central spindle coordinately contribute to spindle stability, we wanted to determine whether kinetochore assembly also depends on the CPC. We found that the KMN complex did not localize after depletion of *aurB*, which encodes the CPC component Aurora B kinase (Fig 1). Interestingly, loss of the CPC in *Drosophila* oocytes results in a complete loss of microtubules around the karyosome (Fig 1) [10]. This phenotype is more severe than the double knockdown of *Spc105R* and *sub* in which ~40% of oocytes showed significant spindle microtubules, albeit thin and disorganized (Fig 6C and 6D). These data demonstrate that while the CPC controls oocyte spindle stability through its regulation of kinetochore assembly and SUB localization, the CPC also regulates additional spindle assembly factors that promote the initiation of spindle assembly.

## Discussion

**Fig 7:**
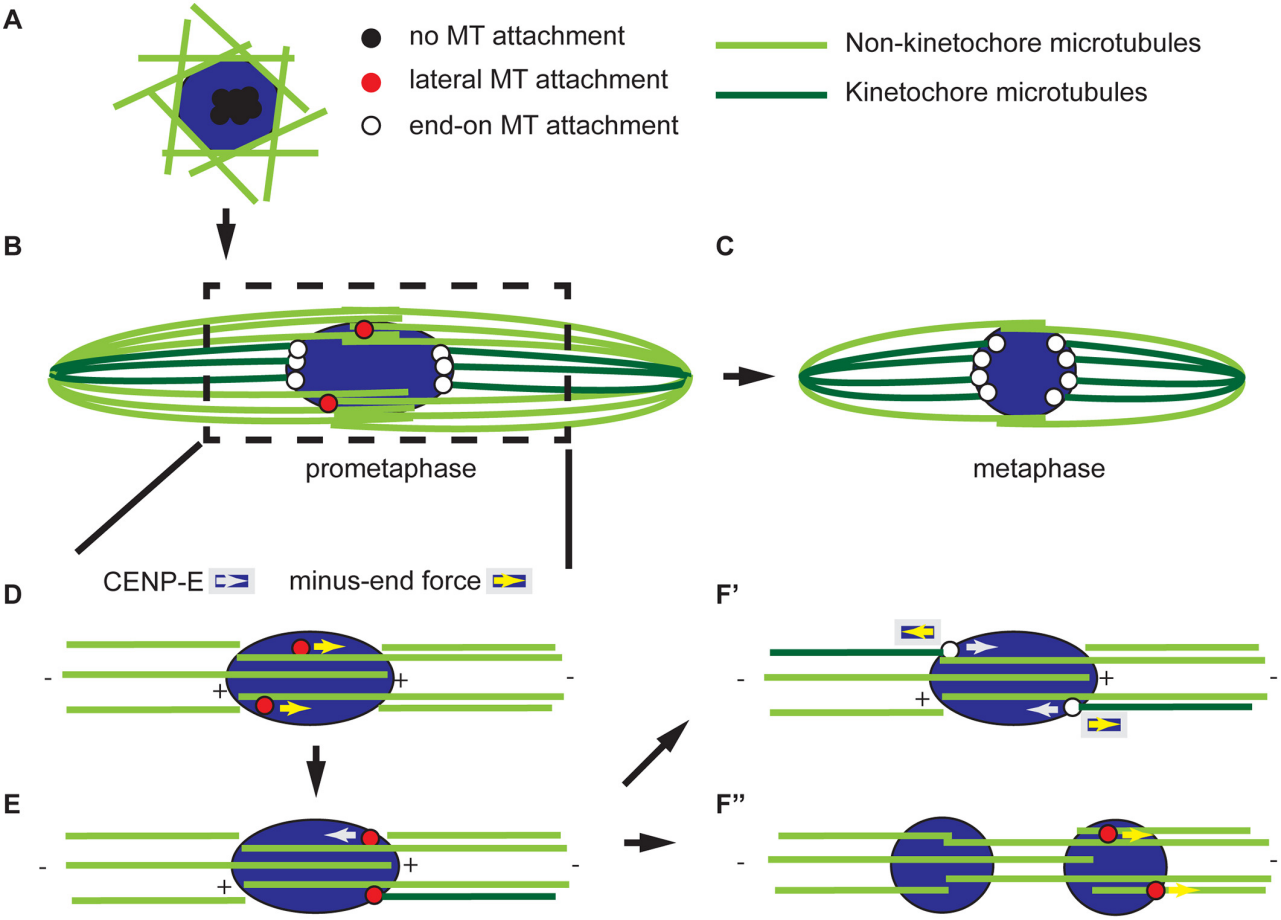
Model for acentrosomal spindle assembly and chromosome orientation. The spindle is composed of microtubules that make end-on attachments to kinetochores (dark green) and those that do not, such as those with plus ends that overlap in the central spindle (light green). (A) Meiotic spindle assembly begins with the accumulation of microtubules around the DNA and the centromeres clustered. These centromeres (shown in black) are not attached to microtubules. (B) Prometaphase is characterized by the presence of kinetochores (red) interacting laterally with microtubules. The process of building a bipolar spindle between panels A and B involves organizing a central spindle and pole-focusing and has been described in detail elsewhere [82, 83]. (C) At metaphase, all kinetochores have end-on attachments (white) and little interaction with the central spindle. Metaphase is also characterized by less reliance on the central spindle for stability. (D-F) A model for bi-orientation showing an enlargement of the spindle around the chromosomes. Kinetochores can move laterally along microtubules in a CENP-E-dependent plus-end direction or a minus-end direction by an unknown force. (D) In this example, two homologous centromeres are moving laterally in the minus-end direction along central spindle microtubules of the same polarity; therefore, they move towards the same pole. (E) This error is detected by an unknown mechanism, leading at least one of the kinetochores to fail to make an end-on attachment and move towards the other pole in a CENP-E dependent manner. (F’) If the kinetochore makes an end-on attachment following this movement, the homologs are bi-oriented. The kinetochores maintain their position by balancing the minus-end force with either the chiasmata or CENP-E. In the absence of CENP-E, errors cannot be corrected, leading to mono-orientation as in (E). (F”) Alternatively, the minus-end force can continue to move centromeres towards the poles, either through a lateral or end-on interaction, resulting in a splitting of the karyosome.

In acentrosomal oocytes, spindle assembly depends on the chromosomes. How the chromosomes can organize a bipolar spindle that then feeds back and drives processes like bi-orientation of homologous centromeres has been unclear. Previously, we demonstrated that the central spindle is required for homolog bi-orientation [10]. Here, we have found that several types of functional chromosome-microtubule interactions exist in oocytes, and that each type participates in unique aspects of chromosome orientation and spindle assembly. We present a model for chromosome-based spindle assembly and chromosome movements in oocytes that highlights the multiple and unappreciated roles played by kinetochore proteins such as SPC105R and NDC80, with implications for how homologous chromosomes bi-orient during meiosis I (Fig 7).

### Lateral and end-on microtubule attachments are required for centromere movement and bi-orientation

While the spindle is assembling and becoming organized, our evidence suggests that the chromosomes undergo a series of movements that ultimately result in the bi-orientation of homologous chromosomes. We found that the separation of clustered centromeres is CPC-dependent [10], but not kinetochore-dependent (Figs 3A and 7A). One possibility is that the CPC-dependent interaction of microtubules with non-kinetochore chromatin drives centromere separation. An alternative is that CPC activity may result in a release of the factors that hold centromeres together in a cluster prior to NEB. A candidate for this factor is condensin, a known target of the CPC, that has been shown to promote the “unpairing” of chromosomes in the *Drosophila* germline [48].

Following separation of clustered centromeres, each pair of homologous centromeres bi-orients by separating from each other towards opposite poles. How bi-orientation is established in acentrosomal oocytes is poorly understood. Previous studies in *C*. *elegans* and mouse oocytes have suggested a combination of kinetochore-dependent and kinetochore-independent (e.g. involving chromokinesins and chromosome arms) microtubule interactions drive chromosome alignment and segregation [23, 49]. We have found that kinetochores play multiple roles, and the process of chromosome bi-orientation can be broken down into a series of chromosome movements that depend mostly on the kinetochores. First, the centromeres make an attempt at bi-orientation (Fig 7D). In *Drosophila* oocytes, this results in the directed poleward movement of centromeres toward the edge of the karyosome and is accompanied by a stretching of the karyosome. Lateral kinetochore-microtubule attachments mediated by SPC105R are sufficient for this initial attempt at bi-orientation. End-on kinetochore-microtubule attachments via NDC80, however, are essential to maintain the bi-orientation of centromeres. Maintenance of centromere bi-orientation is associated with the stable positioning of the centromeres at the edges facing the poles (Fig 7F′).

The lateral-based chromosome movements required for chromosome orientation are probably mediated by the meiotic central spindle, which we previously showed was essential for chromosome segregation [9, 47]. In addition, recent reports in both mitotic and meiotic cells suggest that the initial orientation of chromosomes depends on the formation of a “prometaphase belt” that likely brings centromeres into the vicinity of the central spindle [7]. Therefore, we propose that the initial attempt at bi-orientation occurs during the period when both kinetochores and the central spindle are required for spindle stability (Fig 7B). Then, as the oocyte progresses toward metaphase, and the central spindle decreases in importance, this reflects a trend toward the formation of stable end-on kinetochore-microtubule attachments that, in turn, stabilize the bipolar spindle (Fig 7C). This model is also corroborated by evidence from mouse oocytes that stable end-on kinetochore-microtubule attachments form after a prolonged prometaphase [6].

### CENP-E is required for bi-orientation and maintaining karyosome integrity

Our data demonstrate that some chromosome movements, critical for bi-orientation, are dependent on lateral kinetochore-microtubule attachments. The kinetochore-associated kinesin motor CENP-E is thought to be responsible for chromosome movement along lateral kinetochore-microtubule attachments, resulting in chromosome alignment on the metaphase plate [20]. However, because *Drosophila* meiotic chromosomes are compacted into a karyosome prior to NEB, they do not need to migrate in a plus-end-directed manner to achieve congression and alignment. Instead, centromeres must move toward the poles, perhaps in a minus-end directed manner, to achieve bi-orientation (Fig 7D). Interestingly, we found that CENP-E opposes this minus-end directed movement (Fig 7E) because in the absence of CENP-E, the karyosome split via lateral kinetochore-microtubule attachments (Figs 5 and 7F″). It is not yet clear what mediates the minus-end-directed movement, but the motors Dynein and NCD (the *Drosophila* kinesin-14 homolog) or microtubule flux [50, 51] are prime candidates.

We also observed that CMET is required for the correct bi-orientation of homologous chromosomes (Fig 4C and Table 2). The function proposed in opposing minus-end directed movement may be required for making the correct attachments. As the centromere moves to the edge of the karyosome, CENP-E may not only prevent its separation from the karyosome, but could also force it back towards the opposite pole in cases where the homologs are not bi-oriented (Fig 7E). A similar idea has been proposed for CENP-E in mouse oocytes [52]. Alternatively, CENP-E has a second function in tracking microtubule plus-ends and regulating kinetochore-microtubule attachments [43, 44, 53]. In fact, we found that end-on kinetochore-microtubule stability is affected in the absence of CENP-E (S2 and S5 Figs). Regulating the stability of microtubule plus-end attachments with kinetochores is critical for establishing correct bi-orientation of homologs [54]. Therefore, both functions of CENP-E could contribute to the correct bi-orientation of centromeres in *Drosophila* oocytes.

### Co-orientation of sister centromeres

Loss of SPC105R has a more severe phenotype than loss of either NDC80 or CENP-E, consistent with a role as a scaffold [36]. It recruits additional microtubule interacting proteins like NDC80 and CENP-E and also recruits checkpoint proteins such as ROD [36]. In analyzing oocytes lacking SPC105R, we discovered another class of factors it may recruit: proteins required for co-orientation of sister centromeres during meiosis I. Co-orientation is a process that fuses the core centromeres and is important to ensure that two sister kinetochores attach to microtubules that are attached to the same spindle pole [22]. Co-orientation could involve a direct linkage between sister kinetochores, as may be the case with budding yeast Monopolin [55] or in maize, where a MIS12-NDC80 linkage may bridge sister kinetochores at meiosis I [56]. In contrast, in fission yeast meiosis I, cohesins are required for co-orientation. Cohesion is stably maintained at the core centromeres during meiosis I but not mitosis, and this depends on the meiosis-specific proteins Moa1 and Rec8 [57]. There is also evidence that Rec8 is required for co-orientation in Arabidopsis [58] and we found that loss of ORD, which is required for meiotic cohesion, also results in a loss of centromere co-orientation (Fig 3A and 3C). Further studies, however, are necessary to determine if cohesins are required for co-orientation in *Drosophila*. Indeed, the proteins and mechanism that mediate this process in animals has not been known. Recently, however, the vertebrate protein MEIKIN has been found to provide a similar function to Moa1 [38]. Interestingly, both Moa1 and MEIKIN depend on interaction with CENP-C, but do not show sequence homology. Thus, *Drosophila* may have a Moa1/MEIKIN ortholog that has not yet been identified. In the future, it will be important to identify the proteins recruited by SPC105R and their targets in maintaining centromere co-orientation and how these interact with proteins recruited by CENP-C. The mechanism may involve the known activity of SPC105R in recruiting PP1, because PP1 has been shown to have a role in maintaining cohesion in meiosis I of *C*. *elegans* [59].

### Integrating homolog bi-orientation with spindle assembly and meiotic progression

Our model for spindle assembly and chromosome orientation raises several important questions for future consideration. The CPC is required for spindle assembly in *Drosophila* oocytes [10] and our results highlight the importance of two CPC targets in homolog bi-orientation. One target is central spindle proteins, possibly through the CPC-dependent recruitment of spindle organization factors such as SUB [10]. The CPC is also required for kinetochore assembly (Fig 1), similar to what has been shown in yeast, human cells, and *Xenopus* [60] and consistent with the finding in human cells that Aurora B promotes recruitment of the KMN complex to CENP-C [61, 62]. It will be important to identify targets of the CPC that drive the initiation of spindle assembly, centromere separation, and bi-orientation. In addition, while we have found that the CPC is required for kinetochore assembly, it is not known if the CPC promotes error correction by destabilizing kinetochore-microtubule attachments [63–65]. The CPC may not promote kinetochore-microtubule detachment during meiosis because of the different spatial arrangement of sister centromeres during meiosis I. Indeed, it is not known what is responsible for correcting incorrect attachments at meiosis I or how they are differentiated from correct attachments.

In prometaphase, the central spindle and kinetochores contribute to spindle stability. Our data suggests that the kinetochores increase in importance as the oocyte progresses to metaphase (Fig 7C), perhaps as a result of the stabilization of end-on kinetochore-microtubule attachments as homologous chromosomes become bi-oriented. However, lateral kinetochore-microtubule interactions demonstrated some resistance to colchicine and allow bivalents to stretch in mouse oocytes [65]. Thus, further studies are necessary to determine if lateral kinetochore-microtubule interactions also confer some stability. Our model also proposes that the transition from prometaphase to metaphase involves a switch from dynamic lateral kinetochore-microtubule interactions to stable end-on kinetochore-microtubule attachments. This transition involves the loss of central spindle microtubules, which occurs regardless of microtubule attachment status. Further studies will be necessary to determine if the prometaphase-to-metaphase transition is developmentally regulated rather than being controlled by the spindle assembly checkpoint. As proposed in mouse oocytes [65, 66], this may contribute to the propensity for chromosome segregation errors in acentrosomal oocytes by closing the window of opportunity for error correction after key developmental milestones have been passed. Finally, one of the most poorly understood features of meiosis is co-orientation of sister centromeres at meiosis I [22, 55]. What SPC105R interacts with to mediate co-orientation will provide the first insights into the mechanism and regulation of this process in *Drosophila*.

## Materials and Methods

### Fly crosses and RNAi

Flies were crossed and maintained on standard media at 25°C. All loci not described in the text are described in FlyBase (flybase.org) [67]. Fly stocks from the Transgenic RNAi Project (TRiP, flyrnai.org) were obtained either directly or from the Bloomington Stock Center. The *cmet* ^*Δ*^ allele was a gift from Byron Williams and Michael Goldberg. The *ord* mutant was *ord* ^*5*^ */ord* ^*10*^ and the *mei-S332* mutant was *mei-S332* ^*1*^ */Df(2R)BSC597*. The GFP-tagged ROD transgenic line was a gift from Roger Karess. RNAi constructs generated by the Transgenic RNAi Project (TRiP) [68] were: *aurB* (previously known as *ial*, GL00202) [10], *Ndc80* (GL00625), *Spc105R* (GL00392 and HMS01548), and *cmet* (GL00404). The *sub* RNAi construct (GL00583) was moved to a 2^nd^ chromosome location through standard *P* element transposition crosses.

To deplete oocyte proteins by RNAi, the expression of a short hairpin RNA is under control of the UAS/GAL4 system [69]. To confine the expression to oocytes, we used *matα4-GAL-VP16*, which is expressed throughout oogenesis after the initiation of meiosis [10, 70]. The long duration of meiotic prophase allows knockdown of gene expression within one cell cycle, thereby eliminating the possibility of confounding effects from going through rounds of aberrant cell division with decreasing amounts of protein. Some *cmet* RNAi experiments used *nanos-GAL4*:*VP16* [71]. When *nanos-GAL4*:*VP16* was used to express short hairpins to *aurB*, *Ndc80*, or *Spc105R*, no oocytes were produced, probably due to the failure of the germline mitotic divisions. In all cases, the females expressing these short hairpins were sterile.

To quantify knockdown of gene expression, late-stage oocytes were collected from females carrying both driver and RNAi construct by mass disruption of abdomens in a blender filled with phospho-buffered saline (PBS). Oocytes were filtered through meshes to remove large body parts and allowed to settle quickly in solution to remove smaller egg chambers. For reverse transcriptase quantitative PCR (RT-qPCR), total RNA was extracted from late-stage oocytes using TRIzol Reagent (Life Technologies). RNA was converted to cDNA using the High Capacity cDNA Reverse Transcription Kit (Applied Biosystems). The qPCR was performed in a StepOnePlus (Life Technologies) real-time PCR system using TaqMan Gene Expression Assays (Life Technologies, Dm01843531_g1 for *cmet*, Dm01838612_g1 for *Ndc80*, Dm01792082_g1 for *Spc105R*, and Dm02134593_g1 for *Rpll140* as control).

### Generation of mutations in two *Drosophila Cenp-E* genes

Previous work has shown that *cmet* ^*Δ*^ mutations cause lethality [41], but no *cana* mutants have yet been reported. To generate mutations of either *cana*, *cmet*, or both genes, we screened for imprecise excision of a *P* element inserted between the *cana* and *cmet* genes (*P{GawB}NP5235*, S4 Fig). The insertion site is 217 bp away from *cana* and 832 bp away from *cmet*. Excisions were tested for viability by crossing to *Df(2L)BSC236*. DNA was prepared from adult flies for viable excisions [72] and screened for deletion by PCR and DNA sequencing. Lethal excisions were crossed to *cmet* ^*Δ*^ to check viability and to *cana* ^*13*^ for PCR screening of *cana* deletion. For excisions that likely deleted both *cana* and *cmet* sequences, embryos homozygous for lethal excision chromosomes were selected over a GFP-tagged 2^nd^ chromosome balancer [73] and DNA for PCR was prepared by the same method as adult flies.

Three *cana* mutants were isolated, but the results reported here focus on *cana* ^*13*^ in which most of the motor domain was deleted (S4 Fig). Previous studies have shown that the motor domain of CENP-E is required for chromosome movement during mitosis [74], suggesting that *cana* ^*13*^ is a null allele. Interestingly, however, there is an alternative transcript predicted for *cana*, encoding a protein that does not contain the motor domain (S4 Fig). Therefore, if there is a non-motor function for *cana*, this may not be affected by *cana* ^*13*^. Hemizygous *cana* mutants (*cana* ^*13*^ */Df(2L)BSC236*) are viable and fertile, and the percentage of *X* chromosome non-disjunction was similar to wild-type controls (0.1%, n = 2266). These results suggest that the motor domain of CANA does not play an essential role in mitosis or meiosis.

We also isolated three *P* element excisions that were lethal when heterozygous with *cmet* ^*Δ*^, and PCR analysis also showed deletion of *cana* sequence, showing that both *cana* and *cmet* were deleted. For our studies, we focused on *Df(2L)Cenp-E* ^*141*^, which will be referred to as *Cenp-E* ^*141*^ (S4 Fig). This mutation deletes sequences encoding part of the CMET motor domain and the entire CANA motor domain, suggesting that it is null for CENP-E function. Although *Cenp-E* ^*141*^ is lethal when heterozygous with either *cmet* ^*Δ*^ or *Df(2L)BSC236*, rare homozygotes can be found. These *Cenp-E* ^*141*^ homozygotes, however, have developmental abnormalities, including no ovary development (in 100% of dissected the females, only rudimentary ovaries composed of predominantly somatic cells were present), consistent with the idea that CENP-E is important for cell division.

To generate oocytes lacking both *Cenp-E* genes, the *Cenp-E* ^*141*^ allele was crossed onto a chromosome bearing an FLP recombination target (FRT) sequence inserted at 40A near the centromere on the left arm of chromosome 2. Females with this recombinant chromosome were crossed in vials to males with a matching FRT chromosome carrying the dominant female sterile mutation *ovo* ^*D1*^ and a heat-shock-inducible FLP recombinase. After 3–4 days, the parents were transferred to new vials and progeny were heat shocked for one hour in a 37°C water bath. Female progeny carrying both FRT chromosomes and the FLPase were selected for examination as germline clones.

### Immunofluorescence and microscopy

In a sample of late-stage *Drosophila* oocytes, three cell cycle stages are present: prophase, prometaphase, and metaphase. Oocytes in prophase are distinguished by the presence of the nuclear envelope. We skewed the proportion of prometaphase vs. metaphase oocytes in fixed samples by controlling the age of the females and speed of egg-laying [18]. Late-stage oocytes were collected either from two- to four-day-old females aged two days on yeast with males (“prometaphase”-enriched) or from three- to thirteen-day-old females aged three to five days on yeast without males (“metaphase”-enriched). Oocytes were prepared for immunofluorescence (5% formaldehyde/heptane fixation) and FISH (8% formaldehyde/100 mM cacodylate fixation) essentially as described [75]. For colchicine experiments, oocytes were incubated for 10 min in 0.12% ethanol (control) or 0.12% ethanol plus 150 μM colchicine prior to fixation.

Primary antibodies used for immunofluorescence were mouse anti-α-tubulin conjugated to FITC (1:50 dilution, clone DM1A, Sigma), rabbit anti-CENP-C (1:5000) [76], rat anti-INCENP (1:400) [77], chicken anti-NDC80 (1:500, Tom Maresca), rabbit anti-NSL1 (1:500) [29], rabbit anti-SPC105R (1:4000) [28], rabbit anti-GFP (1:400, Life Technologies), rat anti-α-tubulin (1:75, clone YOL 1/34, Millipore), and chicken anti-CID (1:250) [78]. Cy3-, Cy5-, AlexaFluor647- (Jackson Immunoresearch), or AlexaFluor488- (Molecular Probes) conjugated secondary antibodies were used. DNA was labeled with Hoechst 33342 (1:1000, Invitrogen) or TO-PRO-3 (1:1000, Invitrogen). FISH probes used were to the AACAC satellite (2^nd^ chromosome) and dodeca satellite (3^rd^ chromosome) as described [14, 75]. Samples were mounted in SlowFade Gold (Invitrogen). Images were collected on a Leica TCS SP5 or SP8 microscope with a 63x, 1.4 NA lens using LAS AF software. Images are shown as maximum projections.

Image analysis was performed with Imaris image analysis software (Bitplane). Spindles and CENP-C foci were identified and distances measured using the Distance Transformation Xtension. XYZ coordinates for CENP-C foci and spindle axes were determined using Imaris. This information was used to calculate angles with respect to the spindle axis using Microsoft Excel.

For live imaging, females were matured at 18°C for 4 to 7 days and oocytes from mature females were manually separated in halocarbon oil (Halocarbon). Oocyte stages 13 and 14 were determined by the morphology of the dorsal appendages [79]. *pUASp-cmet shRNA (TRiP*.*GL00404)attP2/UASp-RCC1*:*mCherry nos-GAL4*:*VP16(MVD1) ROD*:*GFP* and a wild-type chromosome over *UASp-RCC1-mCherry nos-GAL4*:*VP16(MVD1) ROD*:*GFP* were used for a *cmet* RNAi and a control. Imaging was carried out using an Axiovert (Zeiss) microscope attached to a spinning disc confocal head (Yokogawa) controlled by Volocity (PerkinElmer). Structures within oocytes were examined using the Plan Apochromat 63x, 1.4 NA lens (Zeiss). Immersol 518F oil (Zeiss) was applied. Z sections, separated by 0.5 μm, covering the entire fluorescent structure were taken every 1 min.

### Statistical analysis

Statistical tests were performed using GraphPad Prism software. Prometaphase karyosome configurations and spindle stability were compared using Fisher’s exact test. Distances between CENP-C and the spindle, distances between homologous centromeres using FISH, and the number of CENP-C foci per oocyte were compared using a *t* test. CENP-C angles were compared using a Mann-Whitney test.

## Supporting Information

S1 Fig End-on kinetochore-microtubule attachments in the absence of kinetochore components. Confocal images of localization of ROD tagged with GFP in wild-type oocytes and after knockdown of *cmet*, *Ndc80*, or *Spc105R*. DNA is shown in blue, tubulin is shown in green, ROD is shown in red, and CID (the *Drosophila* homolog of CENP-A) is shown in white in merged images. ROD and DNA are also shown in white in single channel images. Scale bars represent 10 μm.
(TIF)

S2 Fig Depletion of *cmet* destabilizes kinetochore-microtubule attachments. (A) Confocal images showing a single frame from live imaging of wild-type oocytes (top) and after knockdown of *cmet* (bottom) at stage 14. In merged images, ROD is shown in green marked by ROD:GFP and DNA is shown in magenta marked by RCC1:mCherry. In single channel images, either ROD or DNA is shown in white. The arrowhead indicates kinetochores that accumulate ROD:GFP. Scale bar represents 10 μm. (B) Graph showing the frequency of oocytes in which at least one kinetochore accumulated ROD:GFP from live imaging of either stage 13 or stage 14 wild-type oocytes or after knockdown of *cmet*. n = 40, 8, 19 and 12 for wild type and *cmet* knockdown at stage 13, and wild type and *cmet* knockdown at stage 14, respectively. Oocyte stages 13 and 14 were determined by the morphology of the dorsal appendages (see Materials and Methods).
(TIF)

S3 Fig Karyosome configurations in wild-type oocytes. Confocal images showing the variety of karyosome configurations observed in prometaphase-enriched collections of wild-type oocytes. The “figure 8” and “figure 8 + 4ths out” configurations shown would be included in the “prometaphase” category shown in Table 1 and S3 Table. DNA is shown in blue and tubulin is shown in green in merged images. DNA is shown in white in single channel images. Scale bars represent 10 μm.
(TIF)

S4 Fig Genomic region of *Drosophila Cenp-E* homologs. The coding regions of the two *Drosophila Cenp-E* homologs are shown as gray arrows. Black boxes represent the motor domain of each homolog. The black triangle shows the insertion point of the *P{GawB}NP5235* transposable element. The shaded region in *cana* shows the sequence encoded by the putative alternative transcript. Lines below the genomic region show the sequence deleted in the *cana* ^*13*^ and *Cenp-E* ^*141*^ alleles.
(TIF)

S5 Fig Chromosome re-orientation depends on CENP-E. (A) Confocal images showing single frames in a series from live imaging of wild-type oocytes (left) and after *cmet* knockdown (right) at stage 13. In merged images, ROD is shown in green marked by ROD:GFP and DNA is shown in magenta marked by RCC1:mCherry. In single channel images, ROD is shown in white. Yellow and white arrowheads indicate kinetochores with ROD:GFP accumulation, which migrate from one side of the karyosome to the other in wild type but not in *cmet* knockdown. Scale bar represents 10 μm. (B) Graph showing the frequency of stage 13 oocytes from wild type or after *cmet* knockdown with at least one kinetochore with ROD:GFP accumulation that changed position during the course of live imaging. n = 13 and 7 for wild type and *cmet* knockdown, respectively.
(TIF)

S1 Table Generation of germline clones. (DOCX)

S2 Table Localization of kinetochore proteins in oocytes after RNAi knockdown of *Ndc80* or *Spc105R*. (DOCX)

S3 Table Metaphase karyosome configurations in the absence of CANA and CMET. (DOCX)
