# Supplementary figures and images for: Lateral and End-On Kinetochore Attachments Are Coordinated to Achieve Bi-orientation in Drosophila Oocytes
Source: PLoS Genet. 2015 Oct 16;11(10):e1005605. doi: 10.1371/journal.pgen.1005605 (PMC4608789; doi:10.1371/journal.pgen.1005605)

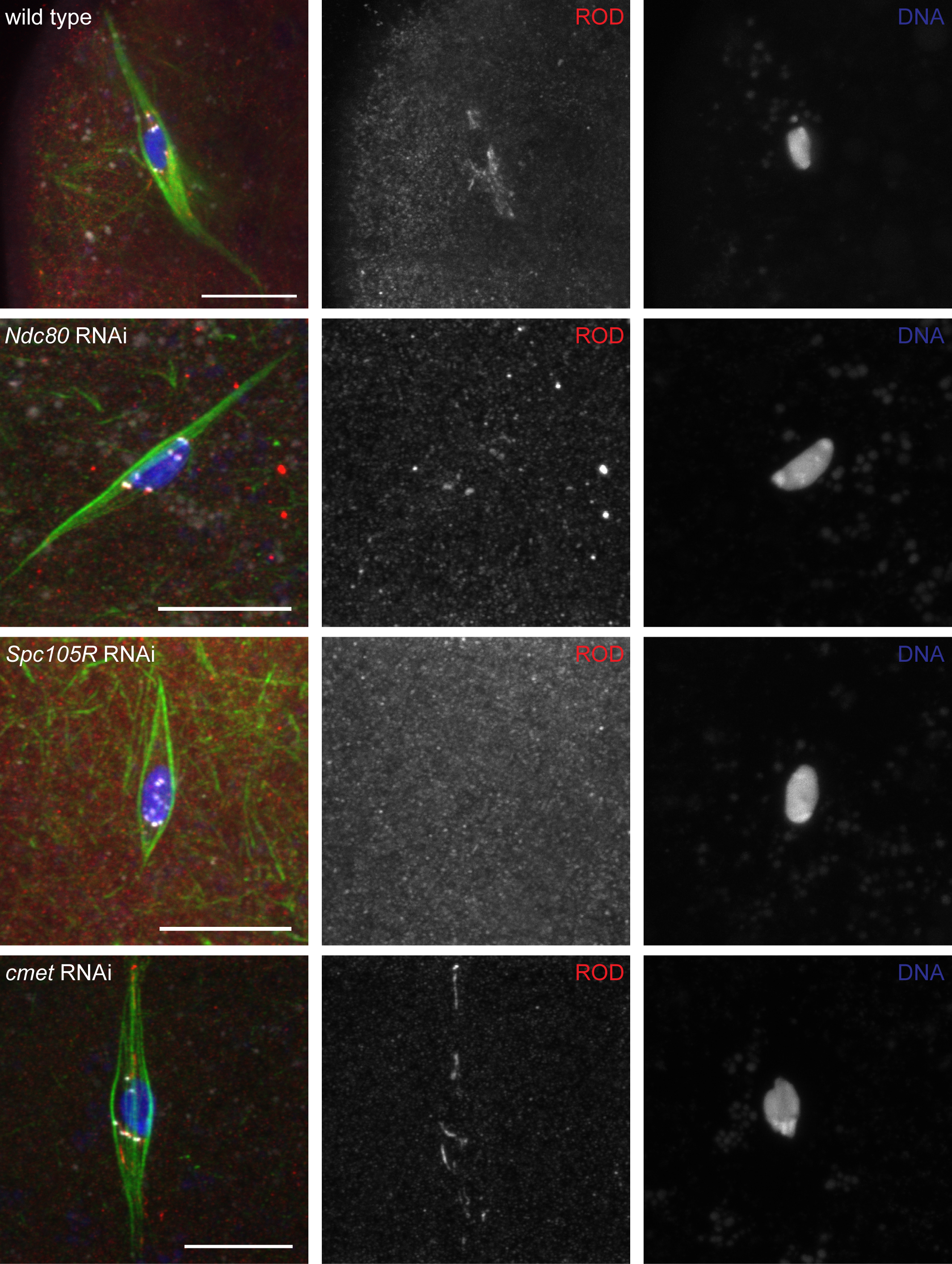

Supplement: S1 Fig — Confocal images of localization of ROD tagged with GFP in wild-type oocytes and after knockdown of cmet, Ndc80, or Spc105R. DNA is shown in blue, tubulin is shown in green, ROD is shown in red, and CID (the Drosophila homolog of CENP-A) is shown in white in merged images. ROD and DNA are also shown in white in single channel images. Scale bars represent 10 μm. (TIF) [file pgen.1005605.s001.tif]

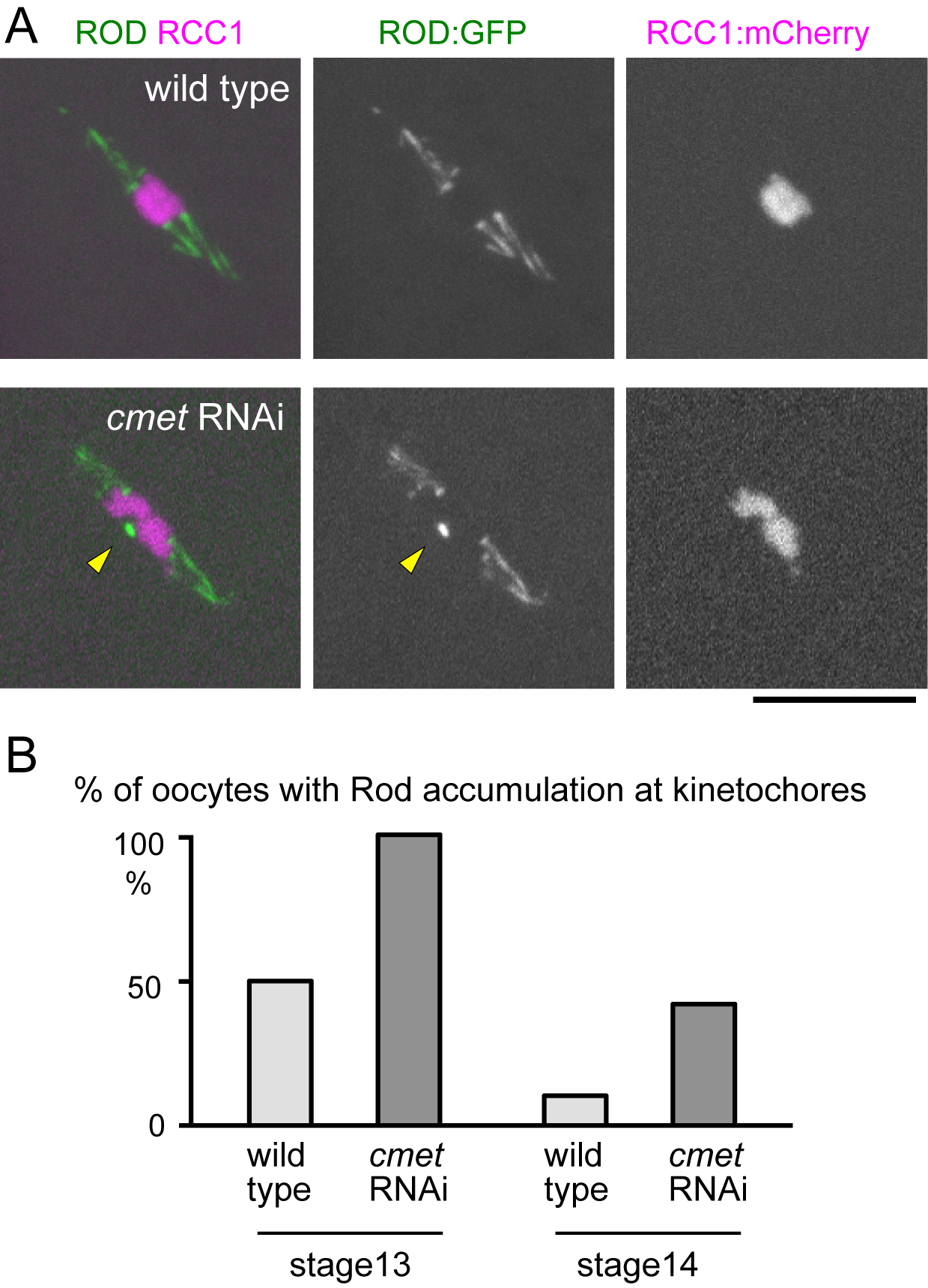

Supplement: S2 Fig — (A) Confocal images showing a single frame from live imaging of wild-type oocytes (top) and after knockdown of cmet (bottom) at stage 14. In merged images, ROD is shown in green marked by ROD:GFP and DNA is shown in magenta marked by RCC1:mCherry. In single channel images, either ROD or DNA is shown in white. The arrowhead indicates kinetochores that accumulate ROD:GFP. Scale bar represents 10 μm. (B) Graph showing the frequency of oocytes in which at least one kinetochore accumulated ROD:GFP from live imaging of either stage 13 or stage 14 wild-type oocytes or after knockdown of cmet. n = 40, 8, 19 and 12 for wild type and cmet knockdown at stage 13, and wild type and cmet knockdown at stage 14, respectively. Oocyte stages 13 and 14 were determined by the morphology of the dorsal appendages (see Materials and Methods). (TIF) [file pgen.1005605.s002.tif]

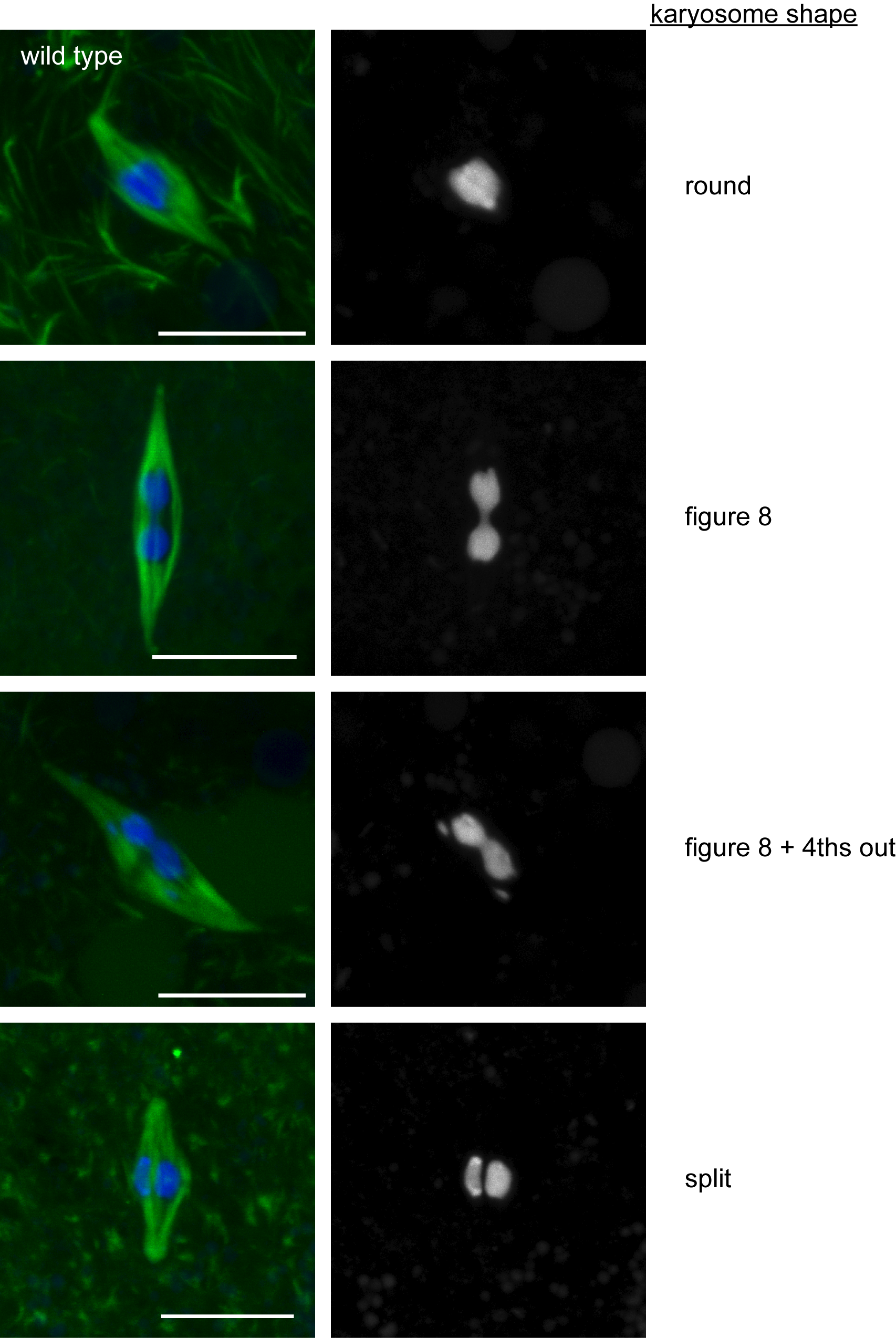

Supplement: S3 Fig — The “figure 8” and “figure 8 + 4ths out” configurations shown would be included in the “prometaphase” category shown in Table 1 and S3 Table. DNA is shown in blue and tubulin is shown in green in merged images. DNA is shown in white in single channel images. Scale bars represent 10 μm. (TIF) [file pgen.1005605.s003.tif]

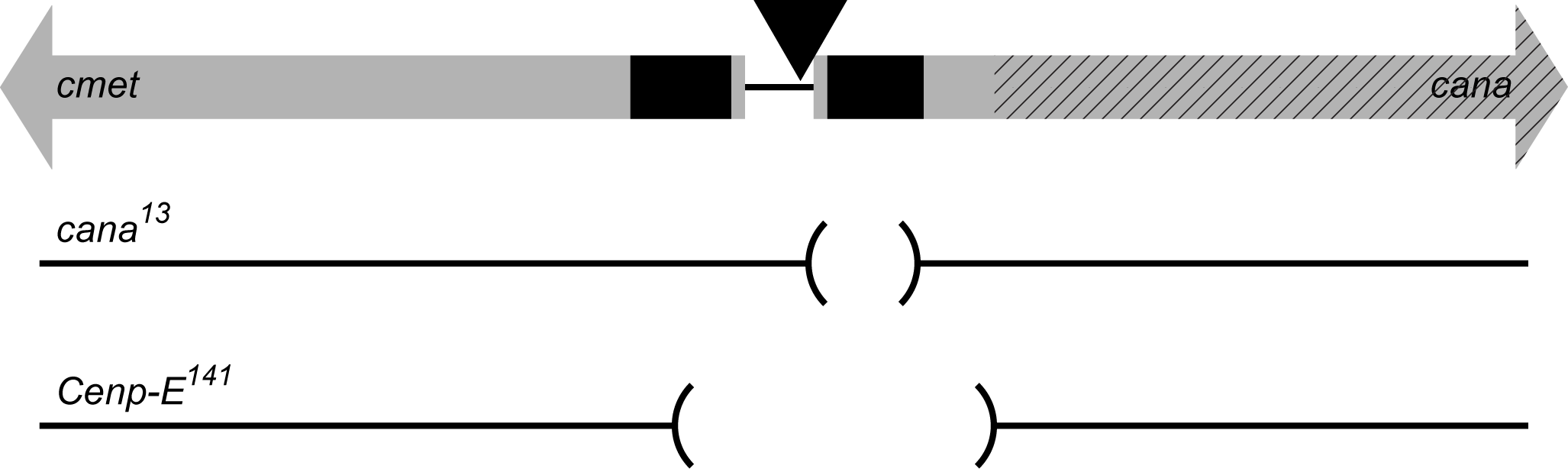

Supplement: S4 Fig — The coding regions of the two Drosophila Cenp-E homologs are shown as gray arrows. Black boxes represent the motor domain of each homolog. The black triangle shows the insertion point of the P{GawB}NP5235 transposable element. The shaded region in cana shows the sequence encoded by the putative alternative transcript. Lines below the genomic region show the sequence deleted in the cana 13 and Cenp-E 141 alleles. (TIF) [file pgen.1005605.s004.tif]

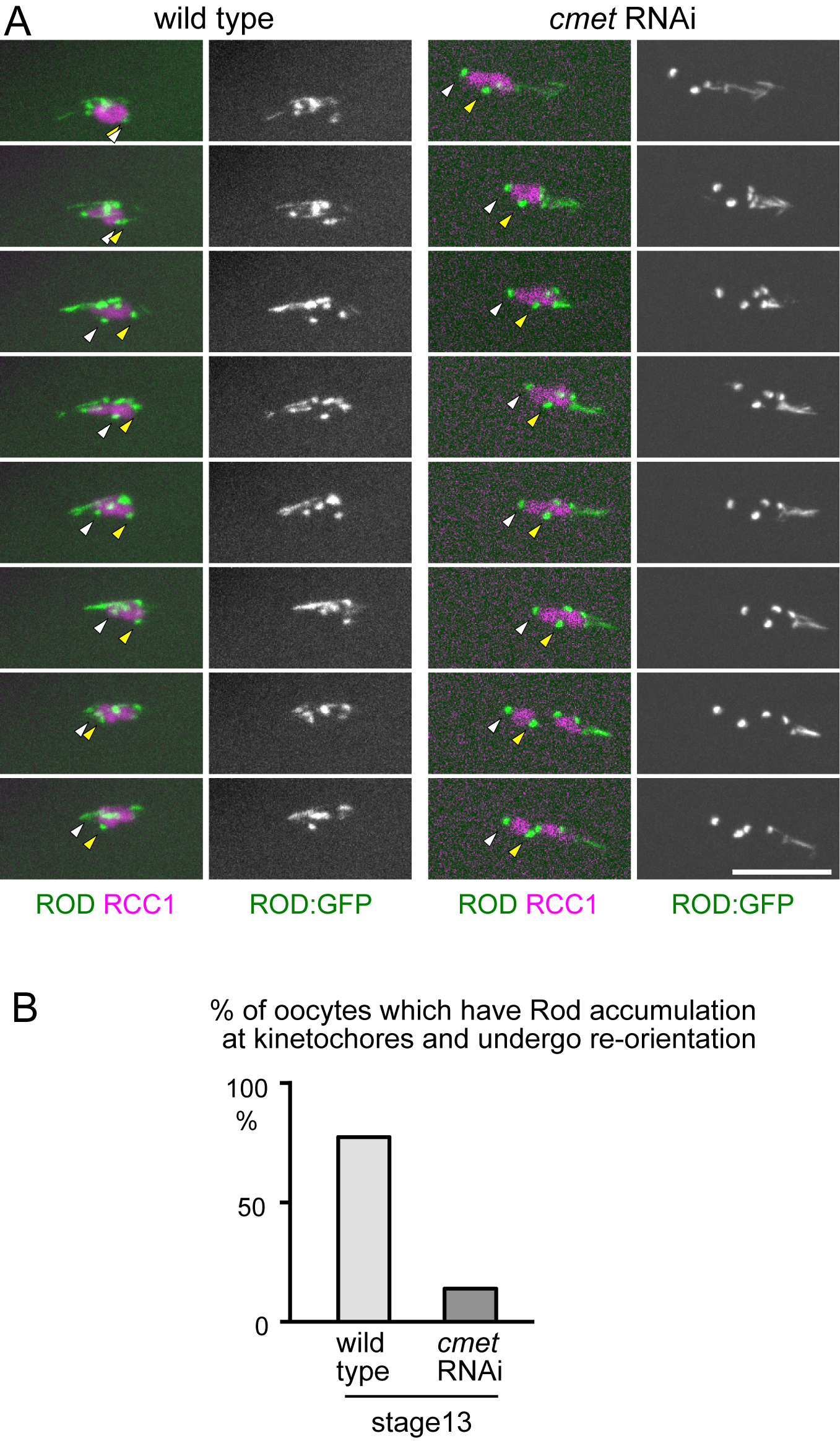

Supplement: S5 Fig — (A) Confocal images showing single frames in a series from live imaging of wild-type oocytes (left) and after cmet knockdown (right) at stage 13. In merged images, ROD is shown in green marked by ROD:GFP and DNA is shown in magenta marked by RCC1:mCherry. In single channel images, ROD is shown in white. Yellow and white arrowheads indicate kinetochores with ROD:GFP accumulation, which migrate from one side of the karyosome to the other in wild type but not in cmet knockdown. Scale bar represents 10 μm. (B) Graph showing the frequency of stage 13 oocytes from wild type or after cmet knockdown with at least one kinetochore with ROD:GFP accumulation that changed position during the course of live imaging. n = 13 and 7 for wild type and cmet knockdown, respectively. (TIF) [file pgen.1005605.s005.tif]
