# Supplementary material for: Lateral and End-On Kinetochore Attachments Are Coordinated to Achieve Bi-orientation in Drosophila Oocytes
Source: PLoS Genet. 2015 Oct 16;11(10):e1005605. doi: 10.1371/journal.pgen.1005605 (PMC4608789; doi:10.1371/journal.pgen.1005605)
Supplement: S1 Table — (DOCX) [file pgen.1005605.s006.docx]

**S1 Table. Generation of germline clones**

| Mutant | No. of females | Females with stage 14 oocytes |
| --- | --- | --- |
| *Mis12* | 42 | 0 |
| *Nuf2* | 104 | 0 |
| *cmet* | 32 | 29 |
