# Supplementary material for: Lateral and End-On Kinetochore Attachments Are Coordinated to Achieve Bi-orientation in Drosophila Oocytes
Source: PLoS Genet. 2015 Oct 16;11(10):e1005605. doi: 10.1371/journal.pgen.1005605 (PMC4608789; doi:10.1371/journal.pgen.1005605)
Supplement: S2 Table — (DOCX) [file pgen.1005605.s007.docx]

S2 Table. Localization of kinetochore proteins in oocytes after RNAi knockdown of *Ndc80* or *Spc105R*

| RNAi | NDC80 | SPC105R | NSL1 |
| --- | --- | --- | --- |
| none | 17/18 | 9/9 | 7/7 |
| Ndc80 | 0/4 | 6/6 | 6/6 |
| Spc105R | 1/13 | 0/4 | 0/6 |
