# Supplementary material for: Lateral and End-On Kinetochore Attachments Are Coordinated to Achieve Bi-orientation in Drosophila Oocytes
Source: PLoS Genet. 2015 Oct 16;11(10):e1005605. doi: 10.1371/journal.pgen.1005605 (PMC4608789; doi:10.1371/journal.pgen.1005605)
Supplement: S3 Table — (DOCX) [file pgen.1005605.s008.docx]

S3 Table. Metaphase karyosome configurations in the absence of CANA and CMET

|  | round | prometaphase^a^ | split^b^ | n | *P^c^* |
| --- | --- | --- | --- | --- | --- |
| wild type | 107 (98%) | 1 (1%) | 1 (1%) | 109 | NA |
| *cana^13^/Df* | 38 (95%) | 2 (5%) | 0 (0%) | 40 | 1.0 |
| *cmet* RNAi | 43 (84%) | 1 (2%) | 7 (14%) | 51 | 0.002 |
| *Cenp-E^141^* | 9 (56%) | 0 (0%) | 7 (44%) | 16 | <0.0001 |

^a^ Prometaphase defined as karyosome in a figure eight shape and/or 4th chromosomes separated from main karyosome mass. See S3 Fig.

^b^ Karyosome is separated into two or more masses of chromosomes

^c^ Fisher’s exact test comparing split/non-split to wild type
